# Comparative phytochemical profiling and bioactivity evaluation of three Indonesian pomegranate (*Punica granatum*) peel varieties: Antioxidant, antibacterial, and in vitro antiproliferative activities

**DOI:** 10.1371/journal.pone.0357371

**Published:** 2026-09-15

**Authors:** Triono Bagus Saputro, Aulia Sunan Fadilah, Anisatus Solihah, Awik Puji Dyah Nurhayati, Panita Chutimanukul, Hamdan Dwi Rizqi

**Affiliations:** 1 Department of Biology, Faculty of Science and Data Analytics, Institut Teknologi Sepuluh Nopember, Surabaya, Jawa Timur, Indonesia; 2 National Center for Genetic Engineering and Biotechnology (BIOTEC), National Science and Technology Development Agency, Khlong Luang, Pathum Thani, Thailand; 3 Department of Chemistry, Faculty of Science and Data Analytics, Institut Teknologi Sepuluh Nopember, Surabaya, Jawa Timur, Indonesia; 4 Advanced Membrane Technology Research Centre (AMTEC), Faculty of Chemical and Energy Engineering, University Teknologi Malaysia, Johor, Malaysia; KTH Royal Institute of Technology, SWEDEN

## Abstract

Antioxidants are compounds that inhibit, prevent, and reduce free radical activity caused by reactive oxygen species (ROS), thereby protecting cells from oxidative damage. Pomegranate (*Punica granatum* L.) is recognized as a valuable source of antioxidants, largely due to the phenolic and flavonoid compounds concentrated in its peel. Variations in cultivar, geographical region, and extraction methods are known to influence its bioactive composition. This study aimed to analyze the antioxidant capacity of the exocarp and mesocarp of three pomegranate varieties (red, yellow, and purple), grown in Indonesia, characterized by their different peel colors, extracted using ethanol (96%) and n-hexane, analyzed separately with total phenolic content (TPC), total flavonoid content (TFC), ABTS, and DPPH assays. The results showed variation in antioxidant capacity and biological compounds depending on both plant tissue and solvent used. Ethanolic extracts generally yielded higher phenolic and flavonoid contents, with the purple exocarp recording the highest TPC (721 mg GAE g^−1^) and TFC (161 mg QE g^−1^). In contrast, n-hexane extracts produce a lower yield, with the red mesocarp as the richest in bioactive compounds, with TPC (70.21 mg GAE g^−1^) and TFC (12.07 mg QE g^−1^). The ethanolic extracts consistently exhibited markedly stronger antioxidant activity, reflected by low IC_50_ values in both ABTS and DPPH assays, with the purple pomegranate exocarp showing the highest potency (IC_50_ value < 50 μg mL^−1^), followed by the red and yellow varieties. In contrast, n-hexane extracts demonstrated substantially weaker activity, indicated by IC_50_ > 200 ppm. Phytochemical profiling through LC-HRMS revealed the presence of diverse polar antioxidants, including phenolic acids, flavonoid glycosides, and anthocyanin derivatives, particularly abundant in the purple peel. Complementary GC-MS analysis confirmed the presence of non-polar constituents such as fatty acids, sterols, terpenoids, and other lipophilic metabolites. The antibacterial activity of the ethanolic peel extracts was evaluated against *Staphylococcus aureus* and *Escherichia coli* using the broth microdilution method, revealing inhibitory effects across all varieties with IC_50_ values ranging from 1417.41 to 5063.08 µg mL^−1^. The ethanolic extract of yellow pomegranate mesocarp exhibited the strongest antibacterial activity, while the ethanolic extract of yellow pomegranate exocarp showed the weakest inhibition. In the MTT assay, all ethanolic extracts of pomegranate peel from three varieties demonstrated antiproliferative effects against Huh7it cells after 48 h incubation. Notably, the purple pomegranate exocarp showed the highest cytotoxic potency (IC_50_ = 231.22 µg mL^−1^). This research can be used as initial information about new sources of antioxidants.

## Introduction

Antioxidants are compounds that hinder the oxidation process by donating electrons to free radical compounds [1]. Free radicals contain one or more unpaired electrons in their outermost atomic orbitals making them highly reactive [2]. This instability encourages free radicals to react with other molecules, damaging cells, proteins, and DNA. In normal cells, the formation and elimination of free radicals are maintained balance, that is disturbed when free radical formation increases, or antioxidant levels decrease. This condition is referred to as “oxidative stress” [3]. An excess of free radicals and oxidative stress are linked to numerous of degenerative diseases, including cancer, heart disease, diabetes, cell necrosis, neurological disorders, dementia, Parkinson’s disease, Alzheimer’s disease, inflammatory diseases, muscular dystrophy, liver disorders, cardiovascular diseases, depression, and aging [4].

Oxidative stress can be effectively neutralized by increasing cell defense through antioxidants. Consuming foods high in natural antioxidants, such as fruits and vegetables, is associated with a reduced risk of degenerative diseases due to oxidative stress, has beneficial nutritional effects, and is considered safe [5]. Antioxidants can also protect materials, especially food, from oxidative damage. Antioxidants are widely used across various industries, including manufacturing, medical, beauty, pharmaceutical, and food industries [6]. Antioxidants are widely used as food preservatives, in cosmetics, and to prevent the degradation of rubber, polymers, and gasoline [7].

The high demand for antioxidants across various industries has driven the antioxidant market value to US$4.0 billion in 2023 and is estimated to reach US$6.5 billion in 2032. In 2023, the antioxidant market was still dominated by synthetic antioxidants [8]. This includes butylated hydroxyanisole (BHA), butylated hydroxytoluene (BHT), tertiary butylhydroquinone (TBHQ), and propyl gallate (PG) [9]. However, the use of this compound may lead to adverse effects due to its characteristics, including carcinogenicity, cytotoxicity, neurotoxicity, induction of oxidative stress and allergic responses, and endocrine disruption [10].

Along with increased attention to the negative impacts of synthetic antioxidants and restrictions on their use, there is a trend towards substituting synthetic antioxidants with natural antioxidants, as they are considered safer [5]. Globally, the agro-food industry produces more than 190 million tons of by-products annually. Processing by-products into value-added products, such as natural antioxidants, can reduce losses, develop sustainable industries, and serve as a source of bioactive compounds for industrial, food, and pharmaceutical applications [11].

One of the agro-food industry waste products that contains many bioactive compounds derived from pomegranates (*Punica granatum* L.) [11]. Pomegranate peel is estimated to contribute approximately 1.6 million tons of global food waste annually [12]. Pomegranate peel is categorized as waste because it is inedible, with sensory and organoleptic characteristics dominated by bitter and astringent tastes, and a hard, dry texture [13]. In fact, pomegranate peel has a higher antioxidant potential than other parts of the fruit, so it can be used as a source of bioactive compounds [14].

Pomegranate (*Punica granatum* L.) is a small tree from the Lythraceae family. Pomegranate fruit has been consumed directly or as juice, jelly, jam, and is widely used as a therapeutic agent [15] to treat heart disease, diabetes, and even as an antioxidant and antimicrobial for food products [16]. Pomegranate peel (*Punica granatum* L.), which comprises about 50% of the fresh fruit weight, has the highest antioxidant potential compared to other parts because it contains the highest concentration of phenolic compounds, with the main content being ellagitannins (ellagic acid, punicalagin, punicalin, and gallic acid), anthocyanins, flavonoids, and tannins. Pomegranate peel also contains lipids such as linoleic acid, palmitic acid, and oleic acid [14].

There are various varieties of pomegranate (*Punica granatum* L.) with various fruit peel colors, ranging from yellow, green, pink, red, to dark purple, which are influenced by the chemical structure and concentration of anthocyanin pigments [13]. In Indonesia, there are three main varieties, red pomegranate, yellow pomegranate, and purple pomegranate [17]. A preliminary study on antioxidants of these Indonesian varieties (red, yellow, and purple) pomegranate peel in Indonesia was conducted by Chasanah in 2020 [18] using the DPPH method on 96% ethanol extracts of red, yellow, and purple pomegranate peel. All three pomegranate varieties exhibit strong antioxidant activity, with purple pomegranate having the most potent antioxidants, followed by yellow pomegranate and red pomegranate.

However, existing global and local investigations on pomegranate peels have several critical limitations. Although numerous studies have demonstrated the antioxidant potential of pomegranate peel, most investigations have focused on a single cultivar, a single extraction solvent, or a limited number of antioxidant assays, making direct comparison among cultivars difficult [14, 18]. Moreover, studies on Indonesian pomegranate varieties remain scarce and are largely restricted to preliminary antioxidant screening, primarily using ethanolic extracts and the DPPH assay [18,19]. Comprehensive information linking phytochemical composition with antioxidant capacity and other biological activities, such as antibacterial and antiproliferative effects, is still lacking, particularly for different peel tissues (exocarp and mesocarp) and extracts with contrasting polarities. Consequently, the phytochemical diversity and biological potential of Indonesian pomegranate peel remain insufficiently characterized despite its promising value as an agro-industrial by-product.

Therefore, this study aimed to comparatively evaluate the antioxidant capacity, phytochemical composition, antibacterial and antiproliferative activities of the exocarp and mesocarp of three Indonesian pomegranate (*Punica granatum* L.) varieties and to characterize the bioactive constituents responsible for their biological activities. By integrating phytochemical profiling with multiple biological assays, this study provides a more comprehensive evaluation of Indonesian pomegranate peel and highlights its potential as a sustainable source of natural bioactive compounds for food, pharmaceutical, and nutraceutical applications.

## Materials and methods

### Plant materials

**Fig 1:**
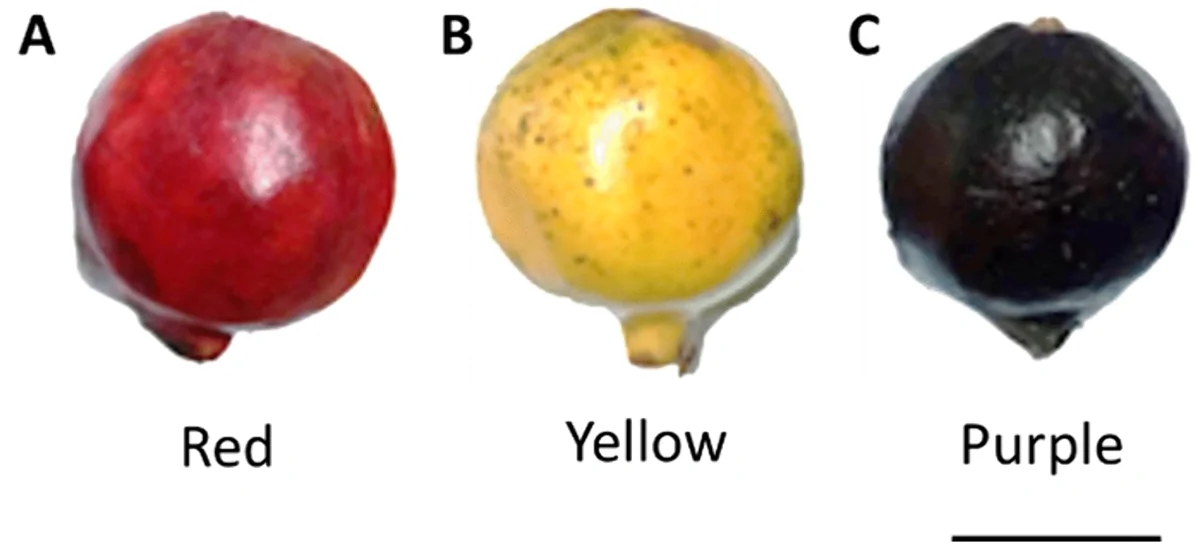
Three varieties of pomegranate grown in Indonesia different in peel color. Bar = 5 cm.

The pomegranates used in this study comprised three varieties distinguished by their peel colors, namely red, yellow, and purple as shown in Fig 1. Fresh fruits were obtained from a local pomegranate farmer and herbal supplier (Yudasaputra Herbal, Situbondo, East Java, Indonesia), where the pomegranate trees are cultivated and harvested under local agricultural practices. Fruits were harvested at the commercial maturity stage and selected based on uniform size, absence of visible physical damage or disease symptoms, and optimal ripening characteristics. According to Cirillo [20], the ripening stage of pomegranates is typically assessed by monitoring exocarp color, juice color, and acidity. Pomegranates are selected based on several criteria, including exocarp color. The exocarp and mesocarp can be distinguished by their harder and thicker texture and color variations. In contrast, the mesocarp is usually white or yellowish-white and tends to be thinner. Accordingly, fruits with fully developed variety-specific peel coloration were selected for this study.

Preparation of pomegranate exocarp and mesocarp extract (*Punica granatum* L.) was carried out after collecting pomegranates. Pomegranates were dry sorted to remove unnecessary parts, cleaned, washed, and peeled [21]. The exocarp and mesocarp of pomegranate fruits (*Punica granatum* L.) were manually separated from the pulp and seeds following the method previously described [22]. Pomegranate exocarp and mesocarp (*Punica granatum* L.) were cleaned by washing with distilled water and dried under shade in a well-ventilated area protected from direct sunlight for approximately seven days. Drying was performed under ambient environmental conditions (average daytime temperature approximately 28–30°C). Plants are dried immediately after collection to avoid damage [21]. After seven days of shade drying, the pomegranate exocarp and mesocarp (*Punica granatum* L.) samples were further dried in a laboratory oven at 30°C for approximately three days (72 hours) until a constant weight was obtained. The dried exocarp and mesocarp were then ground into powder using a grinder, sieved through graduated mesh with 230 and 400 mesh sizes, weighed using an analytical balance, and stored in airtight containers until extraction [18].

### Extraction

After weighing, the pomegranate exocarp and mesocarp powder (*Punica granatum* L.) was obtained by placing it in an airtight glass jar for maceration extraction [23]. Pomegranate Exocarp and mesocarp powder (*Punica granatum* L.), each soaked with solvent (ethanol 96% and n-hexane) separately, with a powder: solvent ratio of 1:20 [24]. The maceration process was carried out for three days (72 hours), and homogenization was carried out every 24 hours by shaking [23].

Each solvent then evaporated naturally using a porcelain cup. The maceration sample was poured into a 300 mL porcelain cup, covered with filter paper, and left to evaporate for 72 hours. After 72 hours, a thick extract of pomegranate exocarp and mesocarp will be obtained and stored in a 10 mL vial bottle [25]. Previously, each thick extract was weighed with an analytical balance, and the percentage yield was calculated using the following equation [26]:


$$\begin{array}{c} Yield\;value=\;\frac{extract\;weight}{weight\;of\;simplicia\;used\;for\;extraction}\;\times \text{1}\text{0}\text{0}\% \end{array}$$

The yield value is closely related to the amount of bioactive content in a sample [26]. Before testing, the crude extracts were diluted. The n-hexane extracts of pomegranate exocarp and mesocarp were diluted with DMSO (dimethyl sulfoxide), and the ethanol extracts of pomegranate exocarp and mesocarp were diluted using distilled water. Dilution of the crude extract was performed by weighing 250 mg of the extract and adding 5 mL of the solvent to produce a concentration of 50,000 µg mL^−1^.

### Experimental design

This study was designed as a comparative phytochemical and bioactivity evaluation of exocarp and mesocarp extracts obtained from three Indonesian pomegranate (*Punica granatum* L.) varieties (red, yellow, and purple). Two extraction solvents with different polarities, namely 96% ethanol and n-hexane, were employed to obtain polar and non-polar phytochemical fractions, respectively. All ethanolic and n-hexane extracts were subjected to total phenolic compound (TPC) and total flavonoid compound (TFC) analyses to compare the phytochemical characteristics among varieties, fruit tissues, and extraction solvents.

The ethanolic extracts were subsequently evaluated for their antioxidant activities using DPPH and ABTS radical scavenging assays, followed by antibacterial activity against the selected bacterial strains (*Staphylococcus aureus* and *Escherichia coli*). Based on the overall phytochemical content and bioactivity, pomegranate exocarp ethanolic extract exhibiting stronger biological activity than mesocarp, therefore it was evaluated for its antiproliferative potential against Huh7it cells using the MTT assay. The ethanolic extract exhibiting the strongest overall bioactivity (purple pomegranate exocarp ethanolic extract) was selected for further characterization. This best-performing extract was subsequently subjected to LC-HRMS to identify polar bioactive constituents potentially associated with the observed biological activities. The same extract also further characterized using proximate analysis to determine its nutritional and compositional baseline.

In parallel, the n-hexane extracts were evaluated for TPC, TFC, and antioxidant activity using DPPH and ABTS radical scavenging assays. The n-hexane extract showing the highest antioxidant performance (red pomegranate mesocarp extract) was selected for GC-MS analysis to characterize its lipophilic chemical constituents. Because GC-MS is more suitable for volatile and non-polar compounds, only the best-performing n-hexane extract was subjected to chemical profiling.

Overall, this workflow was designed to enable a comprehensive comparison of phytochemical composition and biological activities among different pomegranate varieties while allowing detailed chemical characterization of the most bioactive extracts obtained from each extraction solvent.

### Antioxidant capacity

#### Determination of total phenol content.

Total phenolic content (TPC) was determined using the Folin–Ciocalteu colorimetric assay with gallic acid as the calibration standard, adapted from Sukweenadi et al. [27] with minor modifications. A 200 µg mL^−1^ (200 ppm) gallic acid stock solution was prepared by dissolving 2 mg gallic acid in 10 mL of the respective solvent (either deionized water or DMSO). Two distinct sets of working standards were prepared: distilled water was utilized as the solvent for evaluating ethanol extracts, while dimethyl sulfoxide (DMSO) was used for the n-hexane extracts to ensure solubility and matrix compatibility with the samples. Working standard solutions were generated by serial two-fold dilutions of the stock to yield final concentrations of 100, 50, 25, 12.5, 6.25 and 3.125 µg mL^−1^. For each standard, 0.20 mL aliquot of each working standard solution concentration was mixed with 1.80 mL deionized water and 0.20 mL Folin–Ciocalteu reagent (previously diluted 1:1 with water). After incubation at room temperature for 6 min, 2.00 mL of 7% (w/v) Na_2_CO_3_ was added and the reaction mixture was incubated at room temperature for 90 min. Absorbance was measured at 750 nm using a UV–vis spectrophotometer. A calibration curve was constructed by plotting absorbance (y) against gallic acid concentration (x, µg mL^−1^) and fitting a linear regression to obtain the standard equation $\left(y=ax+b,\;R^{\text{2}}\right)$.

Pomegranate exocarp and mesocarp extracts (250 mg mL^−1^ in the extraction solvent) were assayed using the same procedure; 0.20 mL of each extract solution was used per reaction. To eliminate potential background absorbance from the solvent, appropriate solvent blanks containing distilled water or DMSO (without gallic acid or extract) went through the same colorimetric reaction and were used as blank to calibrate the spectrophotometer to zero absorbance. All samples and standards were analyzed in technical triplicate. Total phenolic content was expressed as milligrams gallic acid equivalents per gram of extract (mg GAE g^−1^) using the following formula:


$$\begin{array}{c} C=C_{\text{1}}\times \frac{v}{m} \end{array}$$

C = total phenolic content in mg g^−1^ GAE (Gallic Acid Equivalent); C1 = concentration of gallic acid obtained from the calibration curve in mg mL^−1^; V = extract volume (mL); m = weight of plant extract (grams) [28].

#### Determination of total flavonoid content.

Total flavonoid content (TFC) was determined by the aluminium chloride colorimetric method using quercetin as the calibration standard [27,29]. A 200 µg mL^−1^ (200 ppm) quercetin stock solution was prepared by dissolving 2 mg quercetin in 10 mL of the respective solvent (either deionized water or DMSO). Two distinct sets of working standards were prepared: distilled water was utilized as the solvent for evaluating ethanol extracts, while dimethyl sulfoxide (DMSO) was used for the n-hexane extracts to ensure solubility and matrix compatibility with the samples. Working standards were prepared by serial two-fold dilutions of the stock to yield 100, 50, 25, 12.5, 6.25 and 3.125 µg mL^−1^.

AlCl_3_ 10% reagent was prepared by dissolving 1.5g of AlCl3 with 96% ethanol up to 15 mL. Potassium acetate solution (CH_3_CO_2_K) 1 M was prepared by dissolving 980 mg of potassium acetate (CH_3_CO_2_K) in 10 mL double distilled water (ddH2O). For each standard concentration, 1 mL aliquot of quercetin standard solution was pipetted into a test tube and mixed with 0.2 mL of 10% AlCl3, 0.2 mL of 1M potassium acetate (CH_3_CO_2_K), and 5.6 mL ddH2O. The solution was incubated at room temperature for 30 minutes, and the absorbance was measured at 415 nm using a spectrophotometer. To eliminate potential background absorbance from the solvent, appropriate solvent blanks containing distilled water or DMSO (without quercetin or extract) went through the same colorimetric reaction and were used as blank to calibrate the spectrophotometer to zero absorbance. A calibration curve of quercetin was then made based on quercetin concentration (mg/L) and the resulting absorbance, with the equation where the y-axis represents the absorbance and the x-axis represents the quercetin concentration (x, µg mL^−1^) and fitting a linear regression to obtain the standard equation $\left(y=ax+b,\;R^{\text{2}}\right)$. TFC data are reported in milligrams of quercetin per gram of sample (mg QE g^−1^ sample). TFC in the pomegranate extracts was measured using the same procedure, with 1 mL of extract solution (10 mg mL^−1^ concentration). Each sample was analyzed in technical triplicate, and results were expressed as mg QE g^−1^ extract using the following formula:


$$\begin{array}{c} TFC\;\left(\frac{mgQE}{g}\right)=\frac{C\times V\times F}{m} \end{array}$$

C = sample concentration (mg mL^−1^); v = extract volume (mL); F = dilution factor; m = weight of extract (mg) [28].

#### Determination of DPPH radical scavenging activity.

The 2,2-diphenyl-1-picrylhydrazyl (DPPH) method is widely used to determine the antioxidant capacity of plant extracts and other natural compounds [30]. The antioxidant capacity test process using the DPPH method is divided into two stages: (1) standard preparation using ascorbic acid, and (2) the antioxidant capacity test on pomegranate (*Punica granatum* L.) exocarp and mesocarp with DPPH [29,30].

Ascorbic acid solution is used as a positive control in the DPPH test. A stock solution of 2000 µg mL^−1^ was prepared by dissolving 20 mg of vitamin C in 10 mL of solvent. To ensure solubility and matrix compatibility with the samples, two distinct sets of positive control were prepared: distilled water was utilized as the solvent for evaluating ethanol extracts, while dimethyl sulfoxide (DMSO) was used for the n-hexane extracts. Next, 0.5 mL of stock solution was taken, and 9.5 mL of solvent was added to obtain a concentration of 100 µg mL^−1^. Furthermore, a multi-stage dilution was carried out using the solvent by taking 3 mL of ascorbic acid solution (vitamin C) and adding 3 mL of solvent to obtain the following concentrations: 100, 50, 25, 12.5, 6.25, and 3.125 µg mL^−1^.

DPPH reagent was prepared by dissolving 2 mg of DPPH reagent in 50 mL ethanol 96% to obtain 0.1 mM DPPH reagent. To ensure maximum solubility and matrix compatibility, the crude extracts were dissolved in distinct solvent systems to prepare their respective stock and working concentrations. The n-hexane extracts were dissolved in dimethyl sulfoxide (DMSO) to generate a series of working concentrations at 125, 250, 500, and 1000 µg mL^−1^. Conversely, the ethanol extracts were dissolved in distilled water to obtain working concentrations at 31.25, 62.5, 125, 250, and 500 µg mL^−1^. For the assay, 2 mL of positive control ascorbic acid (vitamin C) solution is pipetted into a test tube, and 2 mL of 0.1 mM DPPH reagent is added. Next, the reagent is vortexed until homogeneous, tightly covered with aluminum foil, and incubated for 30 minutes in a dark place. The solution was then measured for absorbance using a spectrophotometer with a wavelength of 517 nm. DPPH assay for the pomegranate extracts was performed using the same procedure, with 2 mL aliquot of each prepared extract concentration. To eliminate potential background absorbance from the solvent, appropriate solvent blanks containing distilled water or DMSO (without ascorbic acid or extract) went through the same procedure and were used as blank to calibrate the spectrophotometer to zero absorbance. Each sample was analyzed in technical triplicate, and results were expressed as IC_50_. The percentage of free radical destruction was calculated using the following formula:


$$\begin{array}{c} \%Radical\;Scavenging\;Activity=\;\frac{A_{B}-A_{A}}{A_{B}}\times \text{1}\text{0}\text{0} \end{array}$$

AB = absorbance of DPPH reagent

AA = absorbance of sample or standard [2].

The concentration of the extract or standard that shows 50% radical scavenging (IC_50_ value) is obtained from a linear regression of concentration and the percentage of inhibition [2]. The IC_50_ value was calculated according to the regression model used for each extraction system. The IC_50_ value for the n-hexane extracts, IC_50_ values were calculated using linear regression between extract concentration (µg mL^−1^) and percentage inhibition, following the procedure described by [27]. The IC_50_ value was obtained using the following formula:


$$\begin{array}{c} IC\text{5}\text{0}=\;\frac{\text{5}\text{0}-a}{b} \end{array}$$

where *a* is the intercept and *b* is the slope of the linear regression equation relating concentration to percentage inhibition.

For the 96% ethanolic extracts, IC_50_ values were calculated using linear regression between the natural logarithm of extract concentration [ln(concentration)] and percentage inhibition, following the method described by Elmezwghy et al. [2]. The IC_50_ value was therefore calculated as:


$$\begin{array}{c} IC\text{5}\text{0}=\;e^{\left(\frac{\text{5}\text{0}-a}{b}\right)} \end{array}$$

with a and b obtained from the linear regression equation of the relationship between Ln concentration and % inhibition (% RSA) [2,31].

#### Determination of ABTS^+^ radical scavenging activity.

ABTS (2,2’-azino-bis-(3-ethylbenzothiazoline-6-sulfonic) acid), is one of the most widely used methods for antioxidant capacity determination. Antioxidant capacity testing using the ABTS method is divided into three stages: (1) preparation of ABTS reagents, (2) preparation of positive control using trolox solution, and (3) antioxidant capacity testing on pomegranate exocarp and mesocarp (*Punica granatum* L.) with ABTS [30].

ABTS reagent is prepared by mixing ABTS solution and potassium persulfate solution. ABTS solution is prepared by dissolving 0.0406 g of ABTS powder in 10 mL distilled water in a test tube. Potassium persulfate solution is made by weighing 0.007 g of potassium persulfate and then adding distilled water up to 10 mL. Furthermore, 1.5 mL of ABTS solution and 1.5 mL of potassium persulfate solution are homogenized using a vortex and incubated for 12 hours before use. ABTS reagent is made by adding 1 mL of ABTS-potassium persulfate solution and diluting to 50 mL with methanol [32].

Trolox solution is used as a positive control in the ABTS test. To prepare the positive control solution, trolox is diluted with methanol to obtain different concentrations: 100, 50, 25, 12.5, 6.25, and 3.125 µg mL^−1^ through serial dilution. Trolox was prepared in methanol because of its high solubility and stability in this solvent. For each trolox solution concentration, 0.15 mL is taken and put into a test tube, and 2.85 mL of ABTS reagent is added. The solution is then incubated in the dark at room temperature for 30 minutes, and its absorbance is measured at 734 nm using a spectrophotometer. ABTS assay for the pomegranate extracts was performed using the same procedure, with 0.15 mL of extract solution. To ensure maximum solubility and matrix compatibility, the crude extracts were dissolved in distinct solvent systems to prepare their respective stock and working concentrations. The n-hexane extracts were dissolved in dimethyl sulfoxide (DMSO) to generate a series of working concentrations at 125, 250, 500, and 1000 µg mL^−1^. Conversely, the ethanol extracts were dissolved in distilled water to obtain working concentrations at 31.25, 62.5, 125, 250, and 500 µg mL^−1^. Each sample was analyzed in technical triplicate, and results were expressed as IC_50_. The percentage of free radical destruction was calculated using the following formula:


$$\begin{array}{c} \%Radical\;Scavenging\;Activity=\;\frac{A_{B}-A_{A}}{A_{B}}\times \text{1}\text{0}\text{0} \end{array}$$

AB= absorbance of ABTS^+^ reagent

AA = absorbance of sample or standard

The concentration of the extract or standard that shows 50% radical scavenging capacity (IC_50_ value) is determined from a linear regression between the concentration and the percentage of inhibition [2]. The IC_50_ value was calculated according to the regression model used for each extraction system. The IC_50_ value for the n-hexane extracts, were calculated using linear regression between extract concentration (µg mL^−1^) and percentage inhibition, following the procedure described by Sukweenadi et al. [27]. The IC_50_ value can be calculated using the following formula:


$$\begin{array}{c} IC\text{5}\text{0}=\;\frac{\text{5}\text{0}-a}{b} \end{array}$$

where *a* is the intercept and *b* is the slope of the linear regression equation relating concentration to percentage inhibition.

For the 96% ethanolic extracts, IC_50_ values were calculated using linear regression between the natural logarithm of extract concentration [ln(concentration)] and percentage inhibition, following the method described by Elmezwghy et al. [2]. The IC_50_ value was therefore calculated as:


$$\begin{array}{c} IC\text{5}\text{0}=\;e^{\left(\frac{\text{5}\text{0}-a}{b}\right)} \end{array}$$

with a and b obtained from the linear regression equation of the relationship between Ln concentration and % inhibition (% RSA) [2,31].

### Phytochemical content

#### LC-HRMS.

To elucidate the phytochemical profile associated with the highest biological activity, the purple pomegranate exocarp ethanolic extract, which exhibited the highest overall phytochemical content (TPC and TFC) and antioxidant activity (DPPH and ABTS) among all tested samples, was selected for LC-HRMS analysis to characterize its polar bioactive constituents. The chromatographic analysis was performed using a Thermo Scientific™ Vanquish™ UHPLC Binary Pump system equipped with a Thermo Scientific™ Q Exactive™ Hybrid Quadrupole-Orbitrap™ High Resolution Mass Spectrometer equipped with an electrospray ionization (ESI) source. Separation was achieved on a Thermo Scientific™ Accucore™ Phenyl-Hexyl column (100 mm × 2.1 mm ID × 2.6 µm). The mobile phase consisted of eluent A (MS-grade water with 0.1% formic acid) and eluent B (MS-grade methanol with 0.1% formic acid). Chromatographic separation was carried out at a flow rate of 0.30 mL/min using the following gradient program: 95% A/5% B at 0.01 min; 10% A/90% B at 16.00 min; maintained at 10% A/90% B until 20.00 min; and returned to the initial composition (95% A/5% B) at 25.00 min. The injection volume was 3 µL, and the column oven temperature was maintained at 40 °C.

Mass spectrometric detection was performed using an electrospray ionization (ESI) source operated in both positive and negative ionization modes. Nitrogen was employed as the sheath gas (32 AU), auxiliary gas (8 AU), and sweep gas (4 AU). The spray voltage was 3.30 kV, the capillary temperature was maintained at 320°C, and the auxiliary gas heater temperature was 30°C. Full-scan mass spectra were acquired over an m/z range of 66.7–1000 m/z, with a resolving power of 70,000 for full MS acquisition and 17,600 for data-dependent MS/MS (dd-MS^2^) acquisition.

Raw LC-HRMS data were processed using Thermo Scientific™ Compound Discoverer™ version 3.2. Candidate compounds were annotated based on a combination of accurate precursor mass measurements, predicted molecular formula, retention time, peak area, database matching against ChemSpider and mzCloud, and MS/MS spectra obtained through data-dependent acquisition (DDA) when available. Compound annotation was supported by the information generated within the Compound Discoverer workflow, including predicted composition, Δmass (ppm), ChemSpider search, mzCloud search, and spectral matching results. Since authentic reference standards were not employed for compound confirmation, all reported compound identities should be regarded as tentative annotations based on high-resolution mass spectrometric data and database matching.

#### GC-MS.

Based on preliminary phytochemical screening, the red pomegranate mesocarp n-hexane extract, which exhibited the highest phytochemical content (TPC and TFC) and antioxidant activity (DPPH and ABTS) among the n-hexane extracts, was selected for GC-MS analysis to characterize its non-polar phytochemical constituents. The phytochemical analysis of the best performing antioxidant from the n-hexane extract of pomegranate peel was performed using GC–MS. The GC-MS method used is based on research by Yassin, et al. [33]. The phytochemical analysis was performed using the GC–MS Thermo Trace GC Ultra/TSQ Quantum GC mass spectrometer equipped with a TR5-MS capillary column (0.25 µm film thickness × 0.25 mm in diameter × 30 m in length). The analytical conditions were set as follows: pure helium (99.99%) was used as an innert carrier gas at a constant flow rate of 1 mL/min, oven was set to a ramp rate of 6 °C/min to raise the temperature up to 200 °C, injector and detector temperatures were adjusted at 250 °C, the injected volume was 1 µL with split ratio of 1:50. The conditions for spectral mass detection were adjusted as follows: mass range from m/z, 40–400 amu; electron multiplier energy 2000 V; high ionization potential 70 eV. Tentative compound identification of pomegranate mesocarp n-hexane extract was performed by comparing the obtained mass spectra and retention times with entries available in the National Institute of Standards and Technology (NIST) mass spectral library. Since authentic reference standards and retention index confirmation were not employed, all compound identifications were considered tentative.

### Proximate analysis of pomegranate peel

Proximate analysis was performed on the selected sample of the pomegranate peel part with the highest biological capacity. The proximate composition was determined only for the purple pomegranate exocarp, which exhibited the highest overall phytochemical content and biological activity among all tested samples, to provide complementary nutritional information for the extract selected for advanced phytochemical characterization. The analysis was conducted at the Food Engineering Laboratory, Universitas Pembangunan Nasional “Veteran” Jawa Timur, using standardized procedures following SNI 01-2891-1992: Food and Beverage Testing. The parameters analyzed included moisture content, ash, crude fat, crude protein, and carbohydrate. All measurements were conducted in technical triplicate under controlled laboratory conditions.

### Antibacterial activity

The antibacterial activity of the pomegranate peel extracts was evaluated against *Escherichia coli* and *Staphylococcus aureus* using the broth microdilution method with optical density measurement, adapted from the protocol of the Shinrinken Laboratory, Kyushu University to determine the 50% inhibitory concentration (IC_50_). *Escherichia coli* and *Staphylococcus aureus* strains were obtained from the bacterial culture collection of Department of Chemistry, Faculty of Science and Data Analytics, Institut Teknologi Sepuluh Nopember. Based on preliminary phytochemical screening and antioxidant evaluation, the extract polar fraction contained higher levels of phenolic and flavonoid compounds, which are commonly associated with antimicrobial activity. Thus, only the 96% ethanolic extracts were selected for antibacterial testing because they consistently exhibited higher total phenolic content (TPC), total flavonoid content (TFC), and antioxidant activity than the corresponding n-hexane extracts.

Frozen bacterial stocks stored at −80 °C were thawed at room temperature. A total of 50 µL of bacterial suspension was inoculated into 5 mL of sterile Nutrient Broth (NB) under aseptic conditions and incubated at 37 °C for 18 h. The culture was vortexed to ensure homogeneous distribution. For colony isolation, 200 µL of the bacterial suspension was spread onto Nutrient Agar (NA) plates and incubated at 37 °C for 18 h to obtain single colonies. A single isolated colony was transferred into 5 mL NB and incubated at 37 °C with shaking for 18 h. The bacterial density was adjusted spectrophotometrically at 630 nm (OD630). The optical density was standardized to OD630 = 0.4 by diluting the culture with sterile NB, corresponding approximately to 10^9^ CFU mL^−1^ for *Escherichia coli* and 10^8^ CFU mL^−1^ for *Staphylococcus aureus.* Colony counts were verified by serial dilution and plate counting on nutrient agar according to the Shinrinken Laboratory protocol. The ethanolic extracts of pomegranate exocarp and mesocarp are prepared by dissolving extracts in dimethyl sulfoxide (DMSO), to obtain the highest desired stock concentration (e.g., 20 mg mL^−1^). Samples were sonicated to ensure complete dissolution. The final concentration of DMSO in the assay did not exceed 1% (v/v) to avoid solvent-induced antibacterial effects.

The antibacterial activity was determined using a 96-well microplate. For each test, a a master mixture (500 µL) consisting of 445 µL NB, 50 µL standardized bacterial suspension, and 5 µL of the ethanolic extract solution of pomegranate exocarp and mesocarp was prepared. The mixture was gently vortexed. Subsequently, 150 µL aliquots were transferred into individual wells of a sterile 96-well microplate in technical triplicate for each sample. The mixture was gently vortexed and 150 µL aliquots were transferred into microplate wells in triplicate for each sample. Blank wells containing nutrient broth and extract without bacterial inoculum were included to correct for background absorbance from extract colour or turbidity. Negative controls contained NB and bacterial suspension with solvent (DMSO or water), while positive controls contained sorbic acid (2000, 1000, 500 µg mL^−1^) as a reference antibacterial agent. The microplate was sealed and incubated at 37 °C for 18 h. After incubation, bacterial growth was assessed by measuring OD630 using a microplate reader. Antibacterial activity was expressed as percentage growth inhibition relative to the negative control according to the following equation:


$$\begin{array}{c} \%\;Growth\;Inhibition=\;\frac{\left(A_{control}-A_{sample}\right)}{A_{control}}\;\times \text{1}\text{0}\text{0} \end{array}$$

where *A*_*control*_ represents the absorbance of the negative control and *A*_*sample*_ represents the absorbance of the extract-treated culture after blank correction. The concentration required to inhibit 50% of bacterial growth (IC_50_) was determined from concentration–response curves by linear regression analysis using concentration as the predictor y=mx+ c. The IC_50_ value can be calculated using the following formula:


$$\begin{array}{c} IC\text{5}\text{0}=\;\frac{\text{5}\text{0}-c}{m} \end{array}$$

Where *x* represents sample concentration, *y* represents the percentage inhibition (IC_50_ (y = 50)), m is the slope, and *c* is the *y*-intercept of the respective regression equation. All antibacterial assays were performed using three technical replicates, and the results are presented as mean ± standard deviation (SD).

### Cytotoxicity assay (MTT)

Cytotoxicity Assay with MTT Test was performed on the selected sample of the pomegranate peel part extract with the highest biological activity. The cytotoxicity assay was determined only for the pomegranate exocarp ethanolic extract from three varieties, red, yellow, and purple, which exhibited high overall phytochemical content (TPC and TFC) and antioxidant activity (DPPH and ABTS) among all tested samples. Huh7it liver cancer cells were supplemented from Tropical Disease Diagnostic Center, Airlangga University, Indonesia. The Antiproliferative activity of three pomegranate variety exocarp ethanolic extract against Huh7it were appraised using MTT assay. The Huh7it liver cancer cells are cultured in Dulbelcco’s modified Eagle (Invitrogen, Carlsbard, CA, USA) medium enriched with 10% fetal bovine serum (Biowest, Nuaille, France) and Penicillin Streptomycin (SigmaAldrich, St. Louis, MO, USA). Every time cell growth in the petri dish reaches >80%, cell passage is carried out.

Pomegranate exocarp ethanolic extract was tested using MTT [3-(4,5-dimethylthiazol2yl)-5(3-carboxymethoxyphenyl)-2-(4-sulfophenyl)-2H-tetrazolium] assay (Sigma-Aldrich). The cancer cells were pipetted to 96 well plate with density of 2.6x10^4^ cell per well and kept overnight in a 5% CO_2_ incubator at 37°C. 10 mg of crude pomegranate peel extracts were dissolved in 100µl DMSO (stock solution with concentration of 100.000 µg mL^−1^. Then, serial dilution was made from stock solution to obtain a final concentration of 1000 µg mL^−1^, 500 µg mL^−1^, 250 µg mL^−1^, 125 µg mL^−1^, 62,5 µg mL^−1^, 31,25 µg mL^−1^, 15,625 µg mL^−1^, and 7,8125 µg mL^−1^.

The Huh7it cell were treated with the prepared concentration of the pomegranate peel extract. The supernatant was disposed after 48 hours of treatment and 150 µL medium with the developing solution (MTT) (15 µL) was added. The cells were then incubated at 37°C for 4 hours. Finally, the formed formazan crystals were stabilized by adding 100 µL DMSO to the well. The absorbance of the soluble formazan was measured using GloMax Microplate Multidetection Reader (Promega) at wavelength of 560 nm and 750.

The absorbance corresponding to the concentration inducing a 50% inhibition of cell viability (IC_50_) was calculated [33]. Cell viability percentage was obtained by comparing the result of the samples and control. 50% inhibition of the cells was determined using percentage inhibition vs log dose curve.

### Data analysis

All experimental data were expressed as mean ± standard deviation (SD). To evaluate the specific effect of pomegranate varieties on antioxidant activity and total phenolic content (TPC), data were stratified and analysed separately based on the fruit part (exocarp and mesocarp) and the extraction solvent (ethanol and n-hexane). For each stratified subgroup, a One-Way Analysis of Variance (ANOVA) was performed to compare the three Indonesian pomegranate varieties. Duncan’s Multiple Range Test (DMRT) was subsequently applied as the post-hoc test at a significance level of *p* < 0.05 to determine specific significant differences between the varieties within the same tissue and solvent profile. All statistical computations were conducted using IBM SPSS Statistics 27 All experiments were done in technical triplicate, and the data were expressed as the means of triplicate *±* standard deviation (SD).

## Results

### Pomegranate fruit morphology

The pomegranates used in the study were from varieties grown in Indonesia: red pomegranates, yellow pomegranates, and purple pomegranates. The pomegranates from the three varieties used were obtained directly from pomegranate farmers in Situbondo Regency, East Java (Fig 1). The pomegranates used were at an optimal level of ripeness.

Red pomegranate fruit has a diameter of 6–8 cm, with a ruby red exocarp color, and a yellow mesocarp color (Fig 1A). Red pomegranate fruit also has a red aril with a sweet and fresh taste. Yellow pomegranate fruit has a diameter that is the same as red pomegranate fruit, namely 6–8 cm, with a pale yellow exocarp color and a similar mesocarp color (Fig 1B). Yellow pomegranate fruit has a yellow aril with a dry and astringent taste. The purple pomegranate fruit (Fig 1C), has a smaller diameter than the other two varieties, measuring 5–6 cm with a dark purple to blackish exocarp color, and a yellow mesocarp color with a hint of purple. The aril of the pomegranate fruit is pink with a purplish tinge [34].

### Antioxidant capacity of pomegranate peel

Antioxidants are compounds that can prevent, inhibit, and reduce the oxidation process and have the ability to slow down or prevent cell damage caused by free radicals by donating electrons to free radical compounds to stop the oxidation reaction [1]. Both the ethanol and n-hexane extracts of the exocarp and mesocarp of three varieties of pomegranate fruit have an antioxidant capacity indicated by the presence of phenolic compounds, flavonoid compounds, and the ability to reduce ABTS and DPPH reagents. Phenolic and flavonoid compounds are compounds that have a strong correlation with antioxidant capacity because they can capture and stabilize radicals by becoming electron and hydrogen atom donors. These results indicate that higher TPC and TFC correspond to greater antioxidant capacity [35].

#### Total phenolic content.

**Fig 2:**
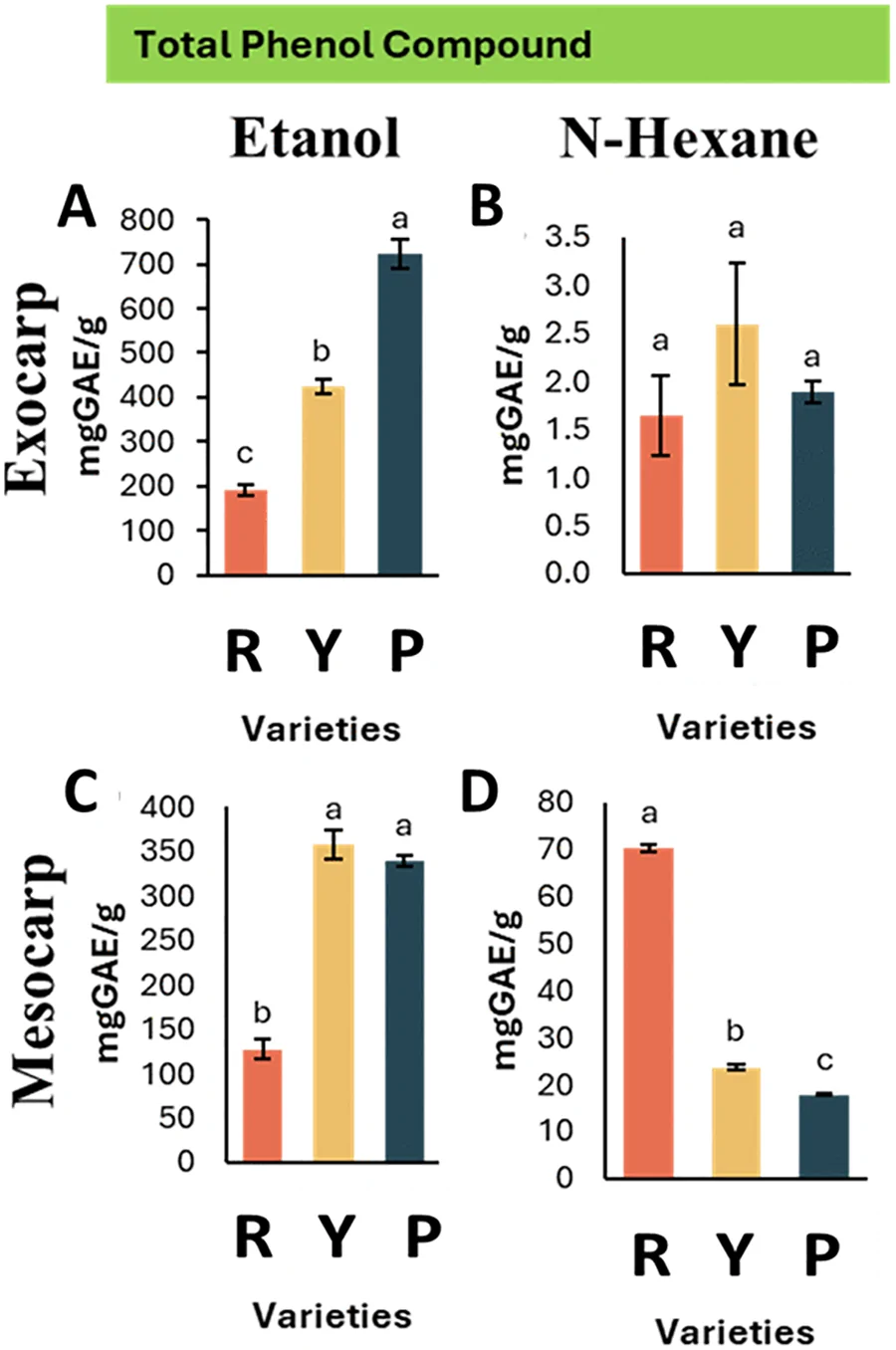
Total phenolic content (TPC) of pomegranate exocarp and mesocarp extracts from three varieties (R = red, Y = yellow, P = purple). (A) Exocarp, ethanol extract; (B) Exocarp, n-hexane extract; (C) Mesocarp, ethanol extract; (D) Mesocarp, n-hexane extract. Values are mean ± SD (mg GAE g^−1^). Different letters above bars indicate significant differences (p ≤ 0.05).

Total phenolic content (TPC) of the pomegranate peel extracts is presented in (Fig 2). Significant differences in TPC were observed among pomegranate varieties, fruit tissues, and extraction solvents (ANOVA, *p* < 0.05). Overall, 96% ethanolic extracts exhibited significantly higher TPC than the corresponding n-hexane extracts. Among the ethanolic extract, purple pomegranate exocarp ethanolic extract revealed the highest phenolic content, recording 721,44 ± 32.939 mg GAE g^−1^.

Fig 2A-D shows the total phenolic content (TPC) of pomegranate extracts showed clear differences among varieties, fruit parts, and solvents. Ethanol extracts consistently yielded markedly higher TPC than n-hexane extracts. In ethanol extracts, the exocarp of the purple variety exhibited the highest phenolic concentration. 721,44±32.939 mg GAE g^−1^ followed by yellow (424,5 ± 15.434 mg GAE g^−1^) and red varieties (192,28± 12.481 mg GAE g^−1^). A similar trend was observed in the mesocarp, with the purple variety showing the highest TPC (338,94± 6.310 mg GAE g^−1^), while the yellow and red mesocarps contained 357,83± 17.220 and 127,56 ± 11.497 mg GAE g^−1^, respectively. By contrast, n-hexane extracts contained substantially lower phenolic levels. In n-hexane extract, the mesocarp shows higher TPC than the exocarp across all varieties, with the red mesocarp recording the highest value (70,21± 1.218 mg GAE g^−1^). Exocarps extracted with n-hexane contained only trace amounts of phenolics, with yellow varieties having a TPC value of 2,6±1.09 mg GAE g^−1^ and the red varieties 1,64±0.71 mg GAE g^−1^. Overall, the results indicate that the exocarp contains higher phenolic compounds than the mesocarp, with ethanol proving to be the more effective solvent.

#### Total flavonoid content.

**Fig 3:**
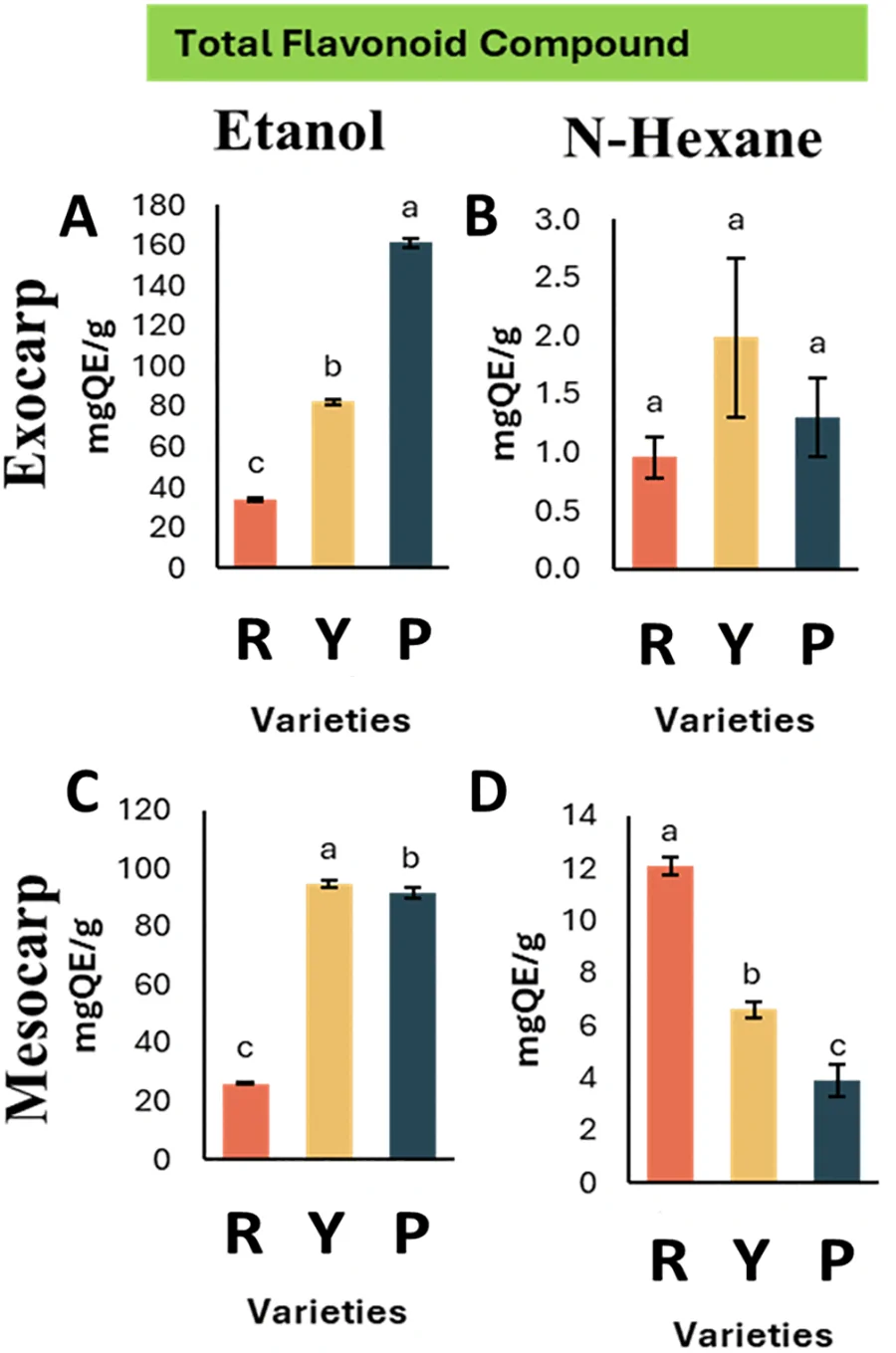
Total flavonoid content (TPC) of pomegranate exocarp and mesocarp extracts from three varieties (R = red, Y = yellow, P = purple). (A) Exocarp, ethanol extract; (B) Exocarp, n-hexane extract; (C) Mesocarp, ethanol extract; (D) Mesocarp, n-hexane extract. Values are mean ± SD (mg QE g^−1^). Different letters above bars indicate significant differences (p ≤ 0.05).

Total Flavonoid Content (TFC) of the pomegranate peel extracts are presented in (Fig 3). The total flavonoid content was tested on the exocarp and mesocarp of pomegranate fruits from three varieties.

The total flavonoid content (TFC) of the pomegranate extracts varied considerably among varieties, fruit parts, and solvents (ANOVA, *p* < 0.05). In general, ethanol extract exhibited markedly higher flavonoid levels compared to n-hexane extract (Fig 3A-D). The highest TFC was observed in the ethanol extract of purple pomegranate (161.33 ± 2.50 mg QE g^−1^), followed by the yellow variety (82.17 ± 1.44 mg QE g^−1^) and the red variety (34.11 ± 0.96 mg QE g^−1^). In the mesocarp, the yellow mesocarp ethanolic extract showed the highest TFC 94.39 ± 1.27 mg QE g^−1^ followed by the purple variety (91.61 ± 1.92 mg QE g^−1^ extract) and the red variety (26.06 ± 0.48 mg QE g^−1^ extract). In contrast, the n-hexane extract yielded significantly lower TFC values than the corresponding ethanolic extracts. Among n-hexane extract, the red mesocarp n = hexane extract presented the highest TFC (12.07 ± 0.59 mg QE g^−1^), followed by the mesocarp of the yellow (6.60 ± 0.51 mg QE g^−1^) and the purple mesocarp (3.86 ± 1.07 mg QE g^−1^). In the exocarp of n-hexane extract, all varieties showed very low flavonoid levels, with the highest found in the yellow variety (1.98 ± 1.19 mg QE g^−1^), followed by the purple (1.30 ± 0.59 mg QE g^−1^) and the red (0.96 ± 0.30 mg QE g^−1^). TFC test on n-hexane extracts of exocarp and mesocarp of three pomegranate varieties in Indonesia produced a higher average concentration of flavonoids in the mesocarp compared to the exocarp.

#### DPPH radical scavenging activity.

**Fig 4:**
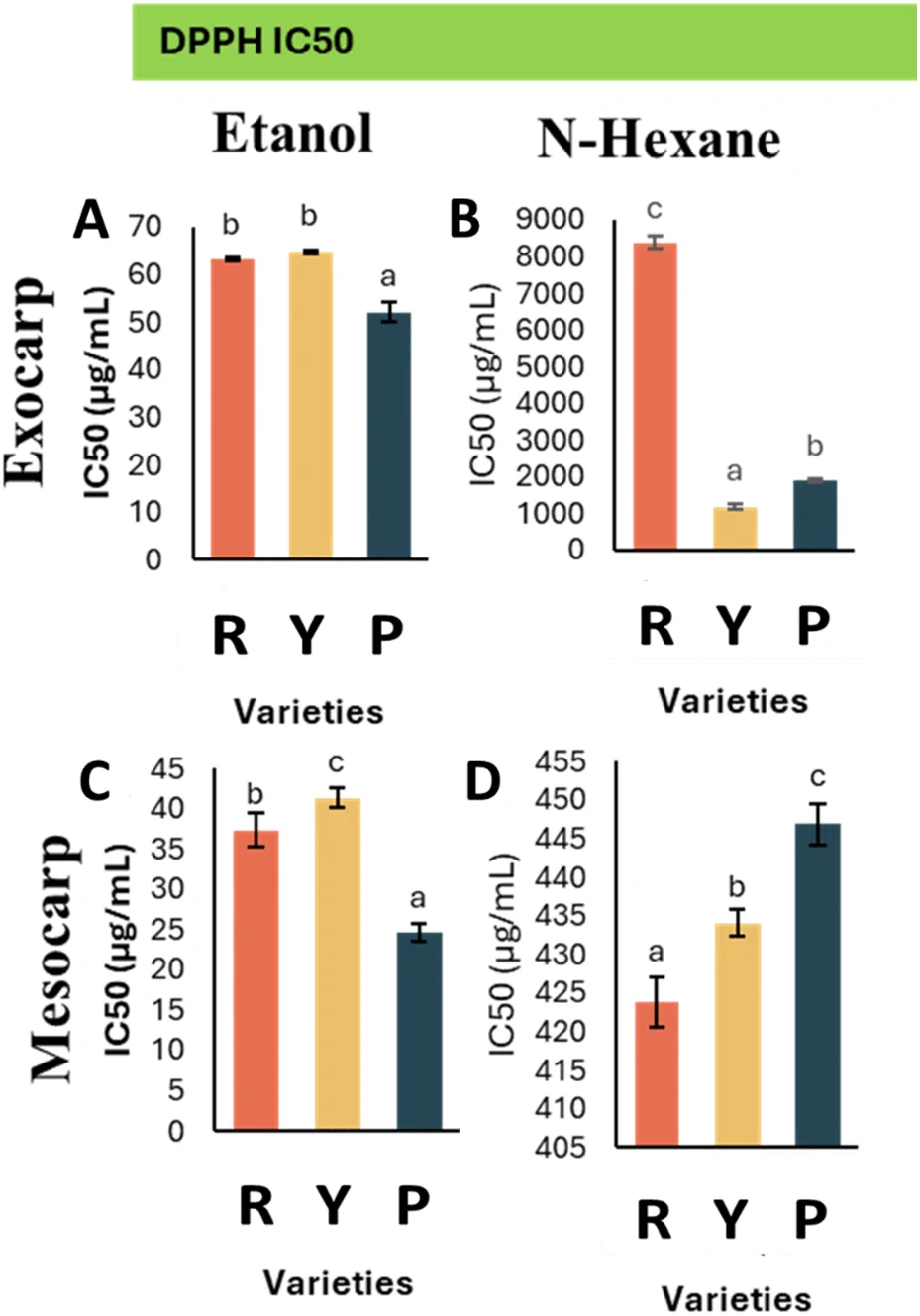
DPPH radical-scavenging activity (IC_50_) of pomegranate exocarp and mesocarp extracts from three varieties (R = red, Y = yellow, P = purple). Panels: (A) Exocarp, ethanol extract; (B) Exocarp, n-hexane extract; (C) Mesocarp, ethanol extract; (D) Mesocarp, n-hexane extract. Bars represent mean IC_50_ (µg mL^−1^) ± SD; different letters denote significant differences (*p* *≤* 0.05).

The DPPH method is performed to determine the antioxidant activity of a sample. The DPPH method was tested on the exocarp and mesocarp of three pomegranate varieties. The DPPH radical scavenging activity of the pomegranate extracts, expressed as IC_50_ is presented in (Fig 4).

The DPPH radical scavenging activity of the pomegranate extracts, expressed as IC_50_ values, showed clear differences among varieties, fruit parts, and solvents (ANOVA, *p* < 0.05). Overall, 96% ethanolic extracts exhibited substantially lower IC_50_ values than the corresponding n-hexane extracts, indicating greater radical scavenging activity. In ethanol extracts, both exocarp and mesocarp exhibited strong antioxidant activity, as indicated by relatively low IC_50_ values (<100 µg mL^−1^) in Fig 4A-D. Among the ethanolic extracts, the purple mesocarp ethanolic extract exhibited the lowest IC_50_ value (24.57 ± 1.17 µg mL^−1^, followed by the red mesocarp (37.35 ± 2.02 µg mL^−1^) and the yellow mesocarp (41.33 ± 1.24 µg mL^−1^). In the ethanolic exocarp, the purple variety also showed the lowest IC_50_ (52.00 ± 1.97 µg mL^−1^), followed by the red (63.10 ± 0.55 µg mL^−1^) and yellow (64.73 ± 0.37 µg mL^−1^) varieties

In contrast, n-hexane extracts displayed much weaker antioxidant activity than the ethanolic extracts, as reflected by markedly higher IC_50_ values. The exocarp showed very low activity with IC_50_ values exceeding 1000 µg mL^−1^. Among the *n*-hexane extracts, the red mesocarp n-hexane extract showed the lowest IC_50_ (423.79 ± 5.80 µg mL^−1^), followed by the white mesocarp n hexane extract (434.05 ± 2.90 µg mL^−1^) and purple mesocarp n-hexane extract (446.84 ± 4.62 µg mL^−1^) Fig 4. The exocarp fractions of n-hexane extract exhibited substantially higher IC_50_ values, with the white, purple, and red varieties recording 1183.05 ± 129.99, 1904.58 ± 87.96, and 8362.92 ± 295.16 µg mL^−1^, respectively. Overall, the results indicate that ethanolic extracts possessed substantially greater DPPH radical scavenging activity than n-hexane extracts, while the purple mesocarp ethanolic extracts, exhibited the strongest antioxidant activity among all tested samples.

#### ABTS radical scavenging activity.

**Fig 5:**
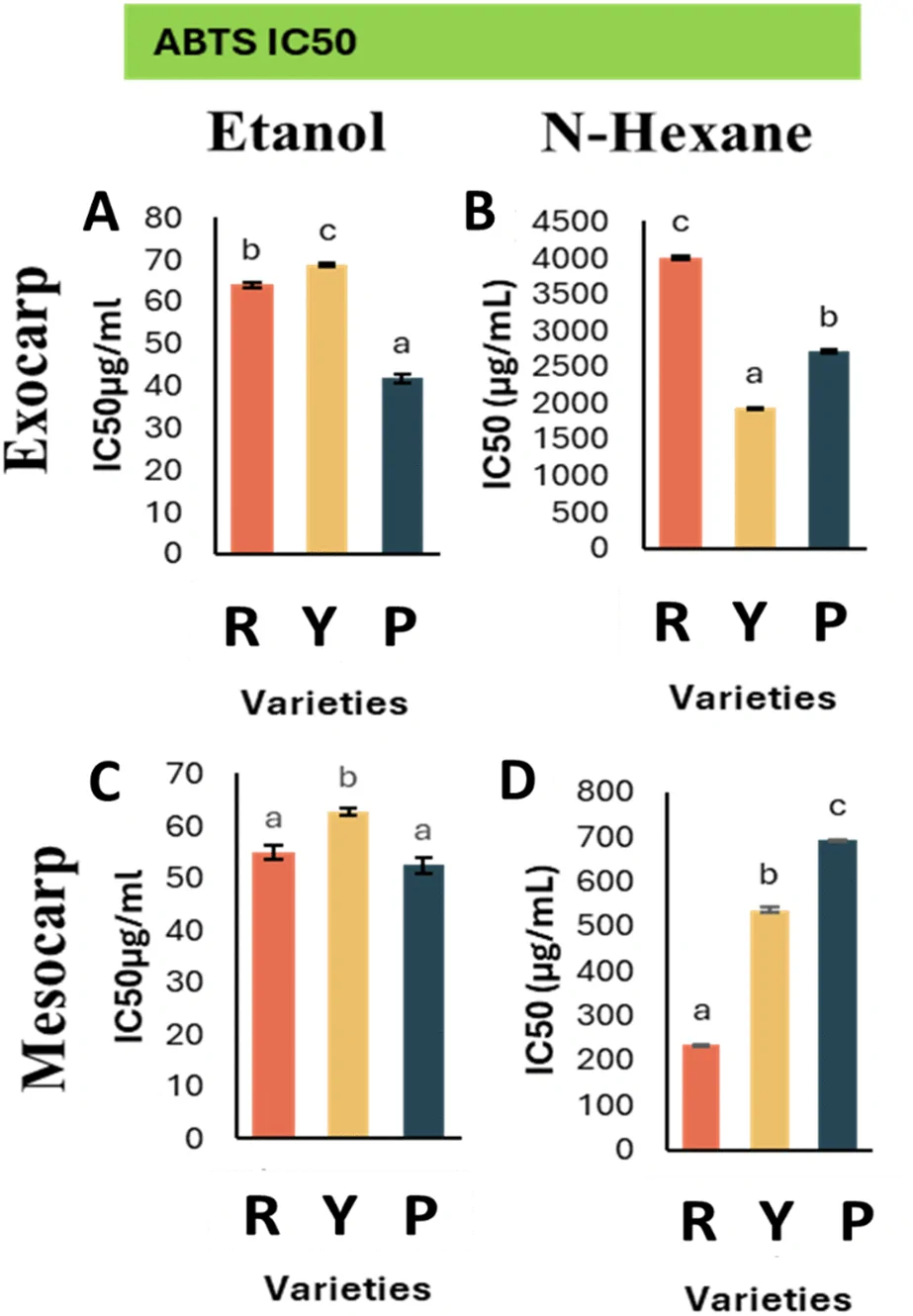
ABTS radical-scavenging activity (IC_50_) of pomegranate exocarp and mesocarp extracts from three varieties (R = red, Y = yellow, P = purple). Panels: (A) Exocarp, ethanol extract; (B) Exocarp, n-hexane extract; (C) Mesocarp, ethanol extract; (D) Mesocarp, n-hexane extract. Bars represent mean IC_50_ (µg mL^−1^) ± SD; different letters denote significant differences (*p* *≤* 0.05).

The antioxidant capacity of pomegranate exocarp and mesocarp extracts expressed as IC_50_ values, varied substantially across varieties and extraction solvents is presented in (Fig 5). Significant differences in antioxidant activity were observed among pomegranate varieties, fruit tissues, and extraction solvents (ANOVA, *p* < 0.05). Overall, 96% ethanolic extracts exhibited substantially lower IC_50_ values than the corresponding n-hexane extracts.

Among the ethanolic extracts, the purple exocarp ethanolic extract exhibited the lowest IC_50_ value (41.726 ± 0.99 µg mL^−1^), followed by the red exocarp (63.931 ± 0.48 µg mL^−1^) and the yellow exocarp (68.770 ± 0.42 μg mL^−1^) (Fig 5A-D). In the mesocarp ethanolic extract, the purple variety also showed the lowest IC_50_ (52.472 ± 1.61 µg mL^−1^), followed by the red (54.857 ± 1.30 µg mL^−1^) and white (62.596 ± 0.71 µg mL^−1^) varieties. Overall, the ethanolic peel extracts demonstrated stronger activity than their mesocarp counterparts.

In contrast, the n-hexane extracts exhibited markedly weaker antioxidant activity (Fig 5A-D). showed by higher IC_50_ values than the ethanolic extracts. Among the n-hexane extracts, the red mesocarp n-hexane extract showed the lowest IC_50_ (234.004 ± 2.28 µg mL^−1^) whereas the red exocarp n-hexane extract exhibited the highest IC_50_ (4007.053 ± 34.60 µg mL^−1^). Similar trends were observed for the yellow and purple varieties, with exocarp extracts generally exhibiting higher IC_50_ values than the corresponding mesocarp n-hexane extracts. The exocarp n-hexane extract showed IC_50_ values of 2701.79 ± 41.37 µg mL^−1^ (purple), and 1926.24 ± 35.98 µg mL^−1^ (yellow). The mesocarp presented IC_50_ values lower than the exocarp. IC_50_ value of the n-hexane mesocarp extract are 234.00 ± 2.28 µg mL^−1^ (red), 535.62 ± 9.14 µg mL^−1^ (yellow), and 690.74 ± 3.79 µg mL^−1^ (purple),

Overall, the results indicate that ethanolic extracts possessed greater ABTS radical scavenging activity than n-hexane extracts, while the purple ethanolic exocarp extract exhibited the lowest IC_50_ among all tested samples.

All categorized as very weak antioxidants [31].

### Antibacterial activity

The antibacterial activity of the tested extracts against *Staphylococcus aureus* and *Escherichia coli* was evaluated for the three varieties of pomegranate exocarp and mesocarp ethanolic extract as it displayed higher phytochemical content and antioxidant activity than n-hexane extracts using the broth microdilution method. The antibacterial efficacy of the extracts was quantitatively evaluated by determining the half-maximal inhibitory concentration (IC_50_). Significant differences in antibacterial activity were observed among pomegranate varieties and fruit tissues (ANOVA, *p* < 0.05).

**Fig 6:**
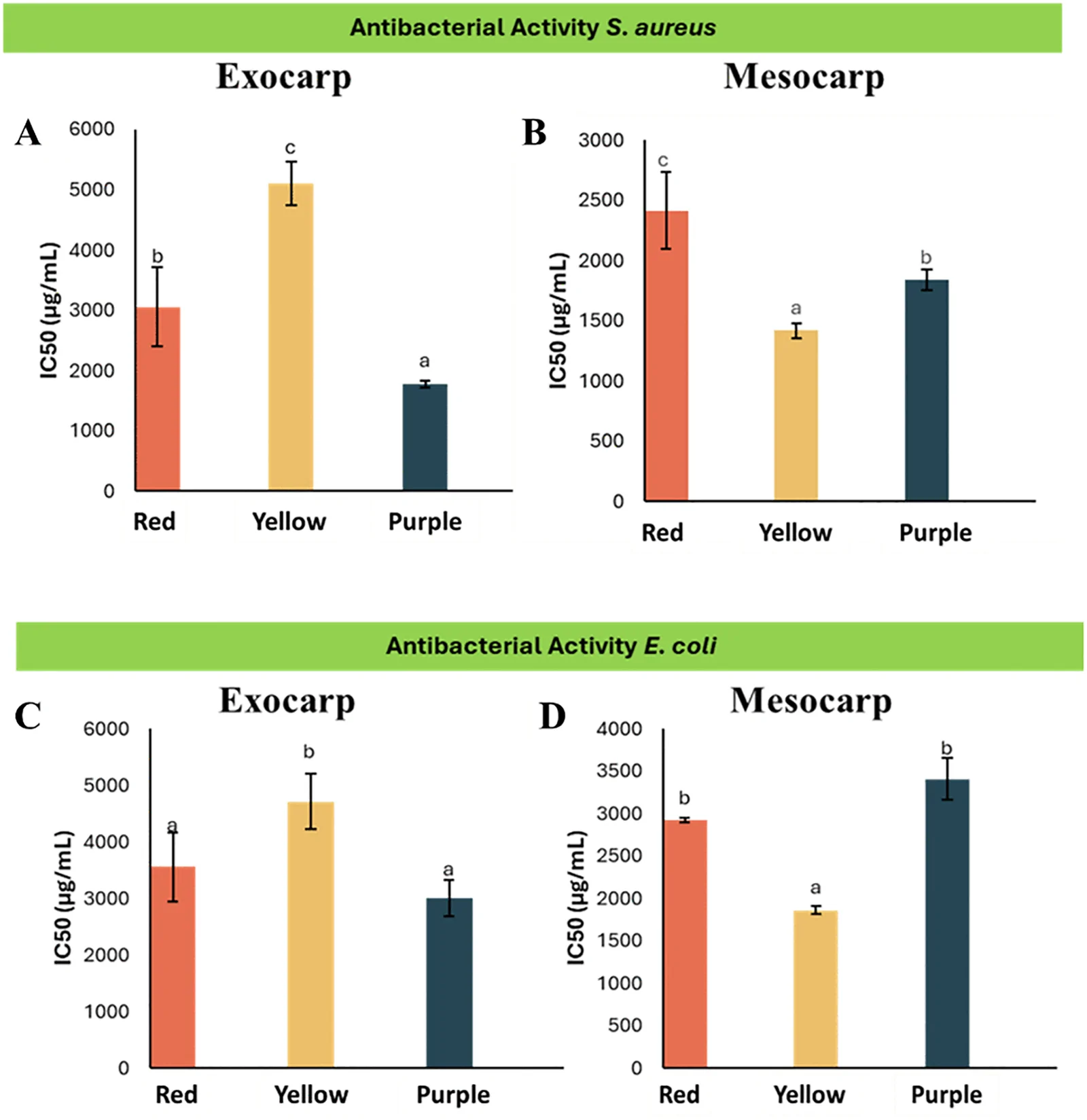
Antibacterial activity of pomegranate peel ethanolic extracts against *Staphylococcus aureus* and *Escherichia coli.* Bar plots show IC_50_ values (µg mL^−1^) for exocarp extracts from red, yellow, and purple varieties; bars are mean ± SD and different letters indicate significant differences (*p* ≤ 0.05). Lower IC_50_ indicates stronger antibacterial activity.

As presented in (Fig 6A-D), all pomegranate exocarp and mesocarp ethanolic extracts exhibited inhibitory effects against both *S. aureus* and *E. coli.* However, the inhibition varied across concentrations and between bacterial species. In general, Gram-positive *S. aureus* showed slightly higher sensitivity compared to Gram-negative *E. coli*, [36]. Against *S. aureus*, the IC_50_ values ranged from 1419.14 ± 61.44 µg mL^−1^ to 5094.05 ± 360.75 µg mL^−1^ (Fig 6A-B). The yellow pomegranate mesocarp ethanolic extract exhibited the strongest antibacterial activity, showing the lowest IC_50_ value (1419.14 ± 61.44 µg mL^−1^), followed by the purple exocarp ethanolic extract (1783.26 ± 55.23 µg mL^−1^) and purple mesocarp ethanolic extract (1837.51 ± 85.54 µg mL^−1^). Intermediate antibacterial activity was observed in the red mesocarp ethanolic extract (2414.78 ± 316.32 µg mL^−1^) and red exocarp ethanolic extract (3053.54 ± 656.66 µg mL^−1^). In contrast, the yellow exocarp ethanolic extract displayed the weakest activity against *S. aureus*, with the highest IC_50_ value of 5094.05 ± 360.75 µg mL^−1^.

A similar pattern was observed against *E. coli* (Fig 6C-D), although higher IC_50_ values were generally recorded. The IC_50_ values ranged from 1863.10 ± 51.22 to 4710.31 ± 492.58 µg mL^−1^. The yellow pomegranate mesocarp ethanolic extract again demonstrated the strongest antibacterial activity, with the lowest IC_50_ value (1863.10 ± 51.22 µg mL^−1^), followed by the red mesocarp ethanolic extract (2922.90 ± 31.24 µg mL^−1^), purple exocarp ethanolic extract (3008.90 ± 321.52 µg mL^−1^), purple mesocarp ethanolic extract (3409.73 ± 241.75 µg mL^−1^), and red exocarp ethanolic extract (3562.35 ± 608.19 µg mL^−1^). The highest IC_50_ value was observed for the yellow exocarp ethanolic extract (4710.31 ± 492.58 µg mL^−1^), indicating the lowest antibacterial activity among the tested extracts.

### Cytotoxicity assay (MTT)

**Fig 7:**
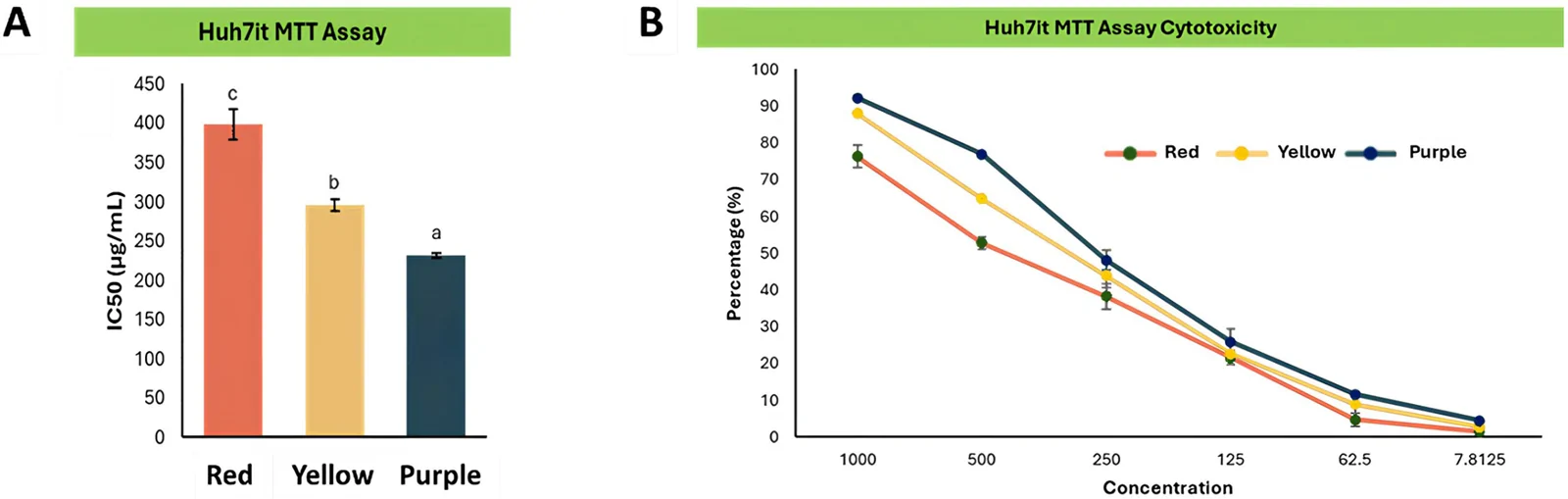
Cytotoxic activity of polar (ethanolic) pomegranate peel extracts against Huh7it hepatoma cells. A. Bar chart of IC_50_ values (µg·mL^−1^) for extracts from red, yellow and purple peels (mean ± SD); B. Dose–response curves from MTT assay showing percent cell viability at increasing extract concentrations for each variety. Lower IC_50_ and steeper decline indicate greater cytotoxic potency.

The cytotoxicity assay of pomegranate peel against cancer cells was examined for the pomegranate exocarp ethanolic extract as it displayed higher antimicrobial and antioxidant activity (Fig 7). The cytotoxic activity of red, yellow, and purple pomegranate exocarp ethanolic extracts against Huh7it cells was evaluated using the MTT assay. Cell viability decreased in a concentration-dependent manner for all tested extracts. Correspondingly, the percentage of cytotoxicity increased as the extract concentration increased. The half-maximal inhibitory concentration (IC_50_) values were calculated from linear regression analysis of percentage cell viability versus extract concentration. Significant differences in cytotoxic activity were observed among the three pomegranate varieties (ANOVA, *p* < 0.05).

The present study demonstrates that all pomegranate extracts exert measurable antiproliferative effects against Huh7it cells, Pomegranate peel extracts cytotoxicity is significantly variable among different extracts (ANOVA, *p* < 0.05). The purple pomegranate exocarp ethanolic extract expressed the highest antiproliferative with the lowest IC_50_ value of 231.22 ± 2.84 µg mL^−1^, followed by the yellow pomegranate exocarp ethanolic extract (295.28 ± 7.44 µg mL^−1^). In contrast, the red pomegranate exocarp extract reveal the lowest potency with IC_50_ of 398.49 ± 18.87 µg mL^−1^. (Fig 7). Post-hoc statistical analysis confirmed that the differences in IC_50_ values among all three varieties were statistically significant (*p <* 0.05). The difference in IC_50_ values indicates variation in cytotoxic potency among variety.

### Phytochemical content

#### Ethanolic 96% extract of purple pomegranate exocarp.

**Fig 8:**
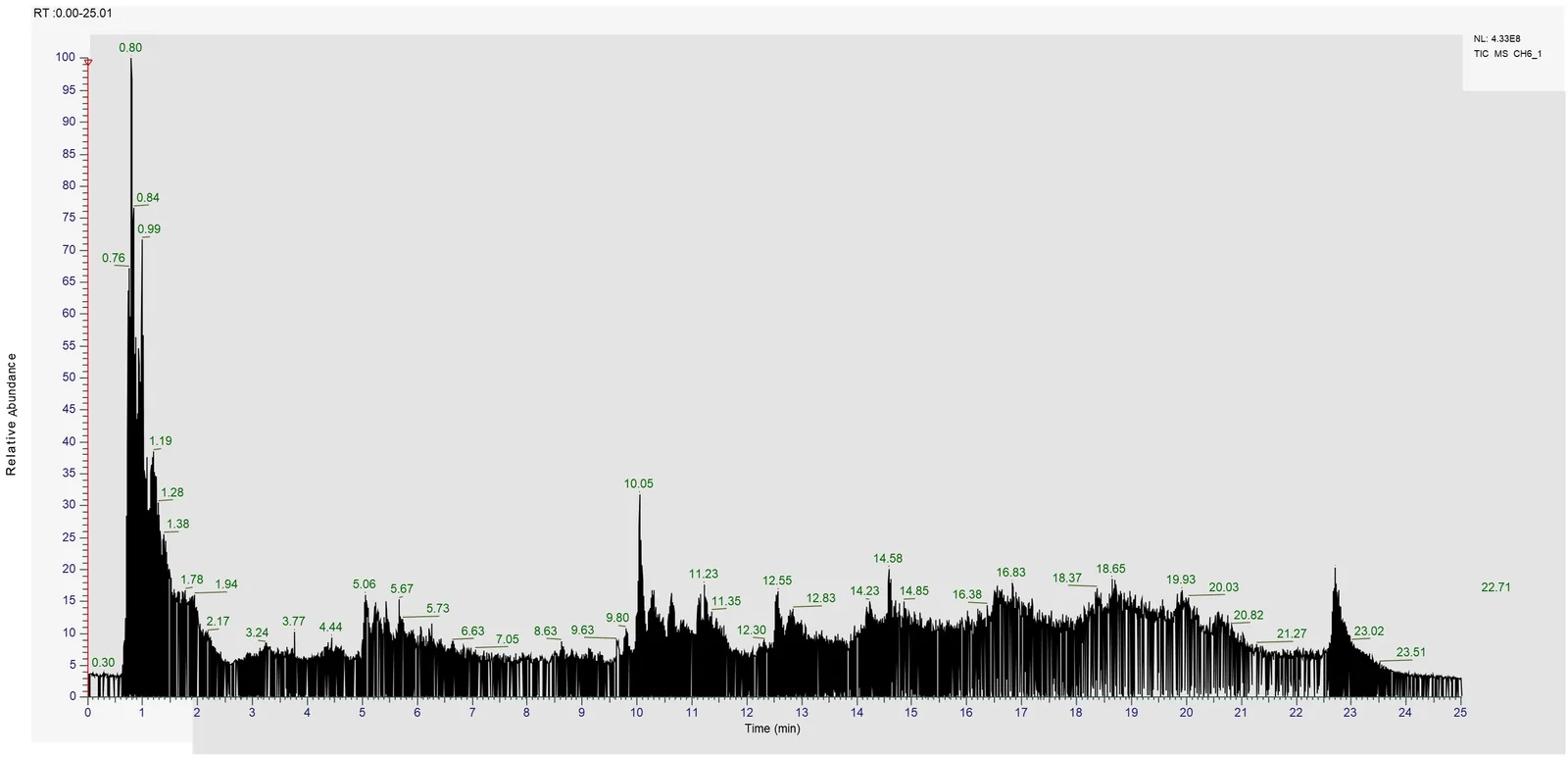
Chromatogram of the purple pomegranate exocarp ethanolic extract obtained by LC-HRMS. The x-axis represents the retention time (min), and the y-axis represents the relative abundance (%).

Since the antioxidant properties and bioactivity tests show that purple varieties have highest performance, we continue to identify the chemical content of pomegranate exocarp ethanolic extract because it exhibited the strongest biological activity among all extracts evaluated. Identification analyses of different phenolic compounds in ethanolic extracts of pomegranate exocarp were performed using LC-HRMS (Fig 8). Compound annotation was conducted using Compound Discoverer 3.2 based on accurate mass measurements and MS/MS spectral matching. As authentic reference standards were not employed, all metabolite identifications should be regarded as tentative. LC-HRMS analysis revealed a chemically diverse metabolite profile comprising organic acids, amino acid derivatives, phenolic acids, flavonoids, glycosides, glycosylated flavonoids, and other secondary metabolites (Table 1). A total of 23 compounds were tentatively annotated. The annotated compounds (largest peak areas) included D-(+)-pyroglutamic acid (≈ 9.7), and several bona fide plant phenolics and flavonoids such as myricetin 3-O-β-D-galactopyranoside, myricetin, quercetin, quercetin-3-β-D-glucoside, ellagic acid, and several additional flavonoid glycosides and phenolic acid derivatives. Among the annotated features, valpromide exhibited the largest chromatographic peak (area ≈12.2). However, because this compound has not been commonly reported as a constituent of *Punica granatum* peel, its annotation was interpreted cautiously as a potential analytical artifact or laboratory contaminant. Excluding this, the chromatographic profile was dominated by several flavonoid and phenolic constituents, including myricetin derivatives, quercetin derivatives, and ellagic acid, together with their corresponding glycosylated forms. These metabolites represented the major annotated phytochemicals detected in the purple pomegranate exocarp ethanolic extract [33].

**Table 1:** LC-HRMS of ethanol 96% extract of purple pomegranate exocarp.

No.	Name	Area
1	Valpromide	12.15
2	D-(+)-Pyroglutamic Acid	9.65
3	TO0127900	7.90
4	Atagabalin	6.40
5	Tripdiolide	5.48
6	6-[(1Z)-1-Propen-1-yl]-2,3,4,5-tetrahydropyridine	5.10
7	NP-000358	3.99
8	6-Propyl-3,4,5,6-tetrahydro-3-pyridinol	3.91
9	myricetin 3-O-beta-D-galactopyranoside	3.45
10	Myricetin	3.27
11	Oleamide	3.17
12	Cianidanol	2.63
13	Choline	2.61
14	Quercetin	2.30
15	Quercetin-3*β*-D-glucoside	1.75
16	1-[(3-Carboxypropyl)amino]-1-deoxy-beta-D-fructofuranose	1.71
17	Methyl isonicotinate	1.48
18	(3*β*,5*ξ*,9*ξ*)-3,23-Dihydroxy-1-oxoolean-12-en-28-oic acid	1.41
19	27USW980DL	1.29
20	Apocynin	1.10
21	D-Glucosamine	0.95
22	(+)-Gibberellic acid	0.86
23	Ellagic acid	0.86

#### n-Hexane extract of red pomegranate mesocarp.

Identification analyses of different phytochemical compounds in n-hexane extracts of pomegranate peel with the highest antioxidant activity were performed using GC–MS. GC–MS analysis was performed on the red pomegranate mesocarp *n*-hexane extract, which was selected for chemical profiling because it exhibited the strongest antioxidant activity among all *n*-hexane extracts evaluated. Compound annotation was carried out by comparison of the acquired mass spectra with the NIST mass spectral library. Since authentic reference standards and retention indices were not employed, all compound identifications should be regarded as tentative. The GC–MS chromatogram revealed a metabolite profile dominated by non-polar and lipophilic constituents (Table 2).

**Table 2:** GC-MS of n-Hexane extract of red pomegranate mesocarp.

No.	Name	Area
1	1-Nonadecene	17.89
2	Cetene	9.82
3	2,4-Di-tert-butylphenol	9.29
4	Octacosane	6.40
5	Dotriacontane	5.63
6	Heptacosane	5.36
7	Tridecanoic acid, 4,8, 12-trimethyl-, methyl ester	2.82
8	1-Tridecene	2.65
9	Tetradecane	2.40
10	Pentadecanoic acid	2.24
11	1-Tridecene	1.81
12	Ethylbenzene	1.77
13	1-Octadecanol	1.63
14	Heptacosane	1.55
15	Heptacosane	1.47
16	1-Octadecanol	1.46
17	1-Pentadecene, 2-methyl	1.19
18	Dotriacontane	1.18
19	1-Pentadecene,	1.12
20	p-Xylene	1.07
21	Oleic acid	0.84
22	1-Octadecanol	0.81
23	1-Nonanol	0.62
24	Dodecane	0.59

GC-MS analysis of the non-polar (n-hexane) extract from the red pomegranate mesocarp revealed 24 compounds tentatively annotated, representing several chemical classes, including hydrocarbons, alkenes, fatty acids, fatty acid derivatives, alcohols, sterols, and phenolic derivatives. The relative abundance of each compound was estimated based on chromatographic peak area percentages. Among the annotated metabolites, 1-nonadecene (area ≈ 17.9%) was the predominant compound, followed by cetene (≈ 9.8%), and 2,4-di-tert-butylphenol (≈ 9.3%), followed by long-chain alkanes such as octacosane and dotriacontane. Other major constituents included octacosane, dotriacontane, pentadecanoic acid, oleic acid, and several additional long-chain hydrocarbons and fatty acid derivatives. Including pentadecanoic acid and oleic acid (trace to low abundance). Likewise, all compound assignments presented in this study remain tentative because they were based solely on mass spectral matching with the NIST library.

### Proximate analysis of purple pomegranate exocarp

The proximate composition of the pomegranate peel with the highest antioxidant capacity revealed its biochemical characteristics. Proximate analysis was conducted on the dried purple pomegranate exocarp powder. This sample was selected for compositional analysis because its corresponding 96% ethanolic extract consistently exhibited the highest biological activity among all pomegranate peel extracts evaluated. The proximate composition of the selected sample is presented in (Table 3). Moisture content was relatively low, indicating minimal residual water and a stable matrix suitable for long-term storage.

**Table 3:** Proximate analysis of purple pomegranate exocarp simplicia.

No	Parameters	Result (%)	Methods
1.	Water content	8.11	Gravimetry
2.	Ash content	3.71	Gravimetry
3.	Protein	6.33	Kjeldahl
4.	Fat	1.03	Soxhlet extraction
5.	Carbohydrate	80.82	By difference

Carbohydrates constituted the major component of the purple pomegranate exocarp, accounting for 80.82% of the total composition. The moisture content was 8.11%, followed by protein (6.33%) and ash (3.71%), whereas crude fat was the least abundant component (1.03%). Overall, the proximate analysis showed that the selected purple pomegranate exocarp was predominantly composed of carbohydrates, with comparatively lower proportions of moisture, protein, ash, and crude fat.

## Discussion

Pomegranate fruit (*Punica granatum* L.) is a plant that not only plays an important role in the food industry but is also used as a traditional medicine and has spiritual value [14]. In Indonesia, there are three main varieties of pomegranate. These varieties are red pomegranate, yellow pomegranate, and purple or black pomegranate [17]. The difference in color of the pomegranate peel is also a characteristic that distinguishes pomegranate varieties because the intensity of the color in pomegranates is influenced by the chemical structure and concentration of anthocyanin pigments in each variety [13]. In addition, pomegranate varieties can also be distinguished by their taste and texture. Red pomegranate has a sweeter and fresher taste, yellow pomegranate has a chewier and rougher texture and a less sweet taste, while black pomegranate has a sweeter taste than red pomegranate [18]. These three varieties also have differences in the structure of their fruit peel. Pomegranate peel consists of exocarp, mesocarp, and endocarp layers. The exocarp layer of black pomegranate consists of 5 layers of sclerenchyma tissue, while in red and yellow pomegranates there are 4 layers of sclerenchyma tissue [17].

Pomegranate (*Punica granatum* L.) has been consumed directly and is widely used as a therapeutic agent, antioxidant and antimicrobial [37]. Pomegranate peel (*Punica granatum* L.), which comprises about 50% of the fresh fruit weight, has the highest antioxidant potential compared to other parts [14] and has been shown to have antioxidant properties because it contains bioactive compounds that can neutralize free radicals such as phenolic and flavonoid compounds [14].

Phenolic and flavonoid compounds are compounds that have a strong correlation with antioxidant capacity because they have the ability to capture free radicals, inhibit lipid oxidation, and stabilize radicals by becoming electron and hydrogen atom donors. The higher of TPC and TFC concentration, the greater the antioxidant capacity of the plant. and TFC. The higher the total phenolic content in the sample, the higher the total flavonoid content [35,38].

As part of this study, we obtained extract from pomegranate peel exocarp and mesocarp from three pomegranate varieties separately. We determined TPC, TFC as well as their antioxidant capacity. We investigated the antibacterial activity of pomegranate peel extracts with higher antioxidant activity against two bacterial pathogens (namely: *S. aureus* and *E. coli*) and antiproliferative effect against hepatocellular carcinoma (Huh7it). Finally, we performed LC-HRMS, GC-MS, and proximate analysis in the most promising antioxidant extract.

Figs 2-3 shows differences in the total phenolic and flavonoid content values of the ethanol and hexanic extract of pomegranate exocarp and mesocarp. Differences in total phenolic and flavonoid levels can be attributed to variety of the plants, different fruit parts, and variations in the growing conditions of the plants. Environmental factors such as soil composition, temperature, rainfall, and ultraviolet radiation can affect the concentration of phenolic components, including flavonoids [13]. Ethanolic extract shows higher phenolic, flavonoid, and antioxidant activity than hexanic extract (P value < 0.05) [33]. This may be attributed to high content of polar phenolic compounds in pomegranate fruit peel, which are responsible for the antioxidant activity [39]. This study is similar with the findings of extract obtained using polar solvent show higher scavenging activity than those obtained using less polar solvent [33,39].

In this study, *Punica granatum* peel (exocarp and mesocarp) extracted with ethanol consistently showed a higher antioxidant capacity, total phenolic content (TPC), and total flavonoid content (TFC) compared to n-hexane extracts across all three Indonesian varieties. Specifically, ethanolic exocarp accounted for the highest TPC and TFC values and exhibited very strong radical scavenging activities in both DPPH and ABTS assays (P value < 0.05), whereas the n-hexane extracts showed markedly higher IC_50_ values indicative of weak antioxidant activity [31]. These observations align with established findings that polar solvents such as ethanol or hydroethanolic mixtures are superior for extracting phenolic and flavonoid compounds, which strongly correlate with antioxidant capacity [33,39].

The greater antioxidant capacity of the exocarp compared to mesocarp in ethanol extracts may be partly attributed to higher concentrations of colored phenolics such as anthocyanins in the exocarp, consistent with broader research indicating that red or purple pomegranate peel typically contain larger amounts of anthocyanins and related flavonoids, which enhance radical scavenging activities [13,40]. In other study, the peel shows higher antioxidant activity than the mesocarp because bioactive compounds with natural antioxidant properties are predominantly found in the peel of plants [41]. Indeed, anthocyanin-rich peel extracts have been shown to correlate with stronger antioxidant activity due to their structural capacity to stabilize free radicals [42]. In this study, purple exocarp exhibited particularly strong antioxidant responses, reflecting this trend. Literature on anthocyanin distribution in pomegranate also suggests that anthocyanin content varies by variety and is concentrated in the exocarp [13].

In contrast, for the n-hexane extract, the opposite pattern was observed: n-hexane extracts overall displayed lower phenolic yields and weaker antioxidant potential than ethanolic extracts, consistent with their extraction of non-polar constituents such as lipids and long-chain hydrocarbons identified via GC-MS [21,33]. However, when comparing exocarp and mesocarp within the n-hexane extract specifically, TPC, TFC, and antioxidant capacity were found to be higher in the mesocarp than in the exocarp across all three varieties. This finding, together with the opposite pattern observed in the ethanolic extract (exocarp higher than mesocarp), confirms that TPC, TFC, and antioxidant capacity are influenced not only by variety but also by an interaction between the fruit part extracted and the extraction solvent used [43]. The significant differences in TPC, TFC, and antioxidant capacity among red, yellow, and purple pomegranate mesocarp (P value < 0.05) also indicate that differences in varieties and cultivars can cause variation in the content of bioactive and phytochemical compounds found in pomegranate skin. Other factors such as the geographical conditions where pomegranates grow, drying methods, and extraction methods can also affect differences in phenolic compounds among samples, consistent with the literature [14].

Nonpolar fractions generally extract fewer phenolic antioxidants, which are largely polar molecules, explaining why both TPC/TFC and scavenging activities were lower for mesocarp and exocarp in n-hexane. This polarity effect aligns with prior research indicating that phenolic extraction and corresponding antioxidant activities are solvent dependent, with polar solvents outperforming nonpolar ones [33,44]. TPC, TFC, and antioxidant capacity measurements using the ABTS and DPPH methods showed varying results between the exocarp and mesocarp of three pomegranate varieties. The results showed that the content of total phenolic compounds (TPC), total flavonoid compounds (TFC), and antioxidant capacity in the three varieties were found to be higher in the mesocarp of pomegranate fruit compared to the exocarp. The differences in total phenolic compounds (TPC), total flavonoid compounds (TFC) and antioxidant capacity found in n-hexane extracts of exocarp and mesocarp of three pomegranate varieties in Indonesia prove that total phenolic compounds (TPC), total flavonoid compounds (TFC) and antioxidant capacity are influenced by the part of the pomegranate that is extracted [43]. The significant differences between total phenolic compounds (TPC), total flavonoid compounds (TFC), and antioxidant capacity in red, yellow, and purple pomegranate mesocarp also indicate that differences in varieties and cultivars can also cause variations in the content of bioactive and phytochemical compounds found in pomegranate peel. Other differences such as the geographical conditions where pomegranates grow, drying methods and extraction methods can also affect the differences in phenolic compounds in samples and with literature results [14]. The non-polar antioxidant capacity obtained from the exocarp and mesocarp of three pomegranate varieties in Indonesia was lower when compared to the non-polar antioxidant capacity obtained from the petroleum ether extract of local pomegranate peel in Khartoum, Sudan. This was due to the higher IC_50_ value obtained compared to the petroleum ether extract of local pomegranate peel in Khartoum with an IC_50_ value of 128 µg mL^−1^. In addition, the non-polar antioxidant capacity of the exocarp and mesocarp of three pomegranate varieties in Indonesia was also lower when compared to the non-polar antioxidant capacity obtained from the pericarp extract of the PTO8 cultivar pomegranate [45]. However, the non-polar antioxidant capacity obtained from the exocarp and mesocarp of three pomegranate varieties in Indonesia was higher than 6 pomegranate cultivars R19, R26, Cvg-Eve, North, Crab, and Cranberry [46].

Antibacterial assay of ethanolic extract pomegranate peel reveals relatively weak antibacterial activity with IC_50_ value ≥1000 µg mL^−1^, indicating low to moderate antibacterial activity according to commonly accepted criteria for plant extracts [47]. Although the antibacterial potency was relatively low, the observed inhibitory trend suggests the presence of bioactive secondary metabolites that may contribute to bacteriostatic effects at higher concentrations. Plant-derived phenolic compounds, flavonoids, and other lipophilic constituents have been widely reported to disrupt bacterial cell membranes, alter membrane permeability, and interfere with essential metabolic pathways [36]. However, crude extracts often exhibit reduced activity compared to purified compounds due to the complexity of phytochemical matrices and potential antagonistic interactions among constituents. show low antibacterial activity, as indicated by relatively high IC_50_ values (>1000 µg mL^−1^) [47]. Compared with the positive control (standard antibiotic), the extracts demonstrated substantially lower antibacterial potency. However, study by Yassin et al., 2021 revealed that methanolic and hexanic extract of pomegranate peel are highly effective against *S. aureus* and *E. coli*. In this study, gram positive bacterial strains are more susceptible to pomegranate peel extract than gram negative bacteria that aligned [33].

In this study, all three pomegranate peel extracts exhibited measurable antiproliferative activity against Huh7it cells as determined by the MTT assay. Among the tested extracts, the ethanolic purple exocarp extract demonstrated the greatest potency (IC_50_ = 231.22 µg mL^−1^), followed by the yellow (IC_50_ = 295.28 µg mL^−1^) and red extracts (IC_50_ = 398.49 µg mL^−1^). These results indicate that pomegranate varieties, and by extension, phytochemical composition such as anthocyanins and phenolic compounds, may influence cytotoxic activity. The observed variation among cultivars may be attributed to differences in phytochemical composition, particularly phenolic compounds, anthocyanins, flavonoids, and ellagitannins, which are known contributors to anticancer activity [33]. Crude extracts are generally considered to have high in vitro cytotoxic activity if the IC_50_ value is ≤ 20 µg mL^−1^, and based on this criteria, the antiproliferative activity of pomegranate extract in this study is relatively low [36]. Although direct comparisons with Huh7it cells are limited in literature, similar research on pomegranate peel extracts against other cancer lines corroborates these findings. For example, ethanolic peel extracts have been reported to exert selective cytotoxic effects against MCF7 cells with IC_50_ of 15,07 µg mL^−1^ [33].

Chemical profiling in this study was performed on two different extracts using two different instruments, and the results should be interpreted separately rather than as a single combined chemical basis for bioactivity. LC-HRMS was performed on the ethanolic purple exocarp extract, which showed the highest overall antioxidant capacity, while GC-MS was performed on the n-hexane red mesocarp extract, representing the non-polar fraction. Globally, pomegranate peel is consistently identified as a rich source of polyphenolic compounds, particularly ellagitannins (e.g., punicalagin and punicalin), phenolic acids such as ellagic acid and gallic acid, and diverse flavonoids including quercetin and catechin, which collectively contribute to multiple biological effects [48]. High-resolution LC-MS studies on various pomegranate cultivars have identified up to dozens of phenolics, with punicalagin, catechin, and ellagic acid frequently among the most abundant constituents. The purple pomegranate exocarp ethanolic extract, which revealed the highest antioxidant capacity was composed of D-(+)-Pyroglutamic acid and triacetonamine with area of 9,65 and 5,48. Our result does not align with Hanafy, et al. 2021 and Yassin et al., 2021 who proved that 5-Hydroxymethylfurfural as main active component of pomegranate methanolic and ethanolic extract [33, 49]. GC-MS analyses of pomegranate peel extracts in the literature have reported long-chain fatty acids or sterols and oleic acid as significant constituents, which may play supportive roles in overall bioactivity profiles, although their direct anticancer implications are less pronounced compared to phenolic compounds [21,33].

Lastly, the variation observed among Indonesian pomegranate varieties in phenolic content and antioxidant capacity is in line with global studies pointing to significant influences of genotype, geographical origin, peel morphology, and extraction methodology on phytochemical profiles. Differences in TPC and TFC among cultivars likely underlie the divergent antioxidant and biological activities, reinforcing the conclusion that both genetic and environmental factors modulate bioactive composition in pomegranate tissues. As supported by international research, greater phenolic diversity and content in peel tissues generally confer stronger functional activities, reaffirming the consistent role of phenolics in mediating antioxidant and health-related bioactivities.

## Conclusion

This study provides a comprehensive evaluation of the antioxidant capacity and phytochemical composition of the exocarp and mesocarp of three Indonesian pomegranate varieties (*Punica granatum* L.) using both polar (96% ethanol) and non-polar (n-hexane) solvents. The ethanolic extracts consistently exhibited markedly stronger antioxidant activity, reflected by low IC_50_ values in both ABTS and DPPH assays, with the purple pomegranate exocarp showing the highest potency, followed by the red and yellow varieties. In contrast, n-hexane extracts demonstrated substantially weaker activity, consistent with their lower phenolic and flavonoid contents. Phytochemical profiling through LC-HRMS revealed the presence of diverse polar antioxidants, including phenolic acids, flavonoid glycosides, and anthocyanin derivatives, particularly abundant in the purple peel. Complementary GC-MS analysis confirmed the presence of non-polar constituents such as fatty acids, sterols, terpenoids, and other lipophilic metabolites characteristic of n-hexane extracts. The antibacterial activity of the ethanolic peel extracts was evaluated against *Staphylococcus aureus* and *Escherichia coli* using the broth microdilution method, revealing inhibitory effects across all varieties with IC_50_ values ranging from 1417.41 to 5063.08 µg mL^−1^. The yellow pomegranate mesocarp exhibited the strongest antibacterial activity, while the exocarp of the same variety showed the weakest inhibition. In the MTT assay, all extracts demonstrated antiproliferative effects against Huh7it cells after 48 h incubation. Notably, the purple pomegranate exocarp showed the highest cytotoxic potency (IC_50_ = 231.22 µg mL^−1^). Among the varieties, the purple exocarp exhibited the strongest antiproliferative activity, indicating a possible association with its higher phytochemical content. Collectively, these findings support the potential of pomegranate peel as a source of multifunctional bioactive compounds for further pharmacological exploration.

## Supporting information

S1 File Antioxidant capacity data. Supporting data for the antioxidant capacity of pomegranate peel extracts, including total phenolic content (TPC), total flavonoid content (TFC), DPPH radical-scavenging activity, and ABTS radical-scavenging activity of exocarp and mesocarp extracts from the three pomegranate varieties.
(XLSX)

S2 File Antibacterial assay data. Supporting data for the antibacterial activity of ethanolic pomegranate peel extracts against *Staphylococcus aureus* and *Escherichia coli*, including the experimental measurements used to determine antibacterial activity and IC_50_ values.
(XLSX)

S3 File Cytotoxicity assay data. Supporting data for the cytotoxicity assessment of ethanolic pomegranate exocarp extracts from the three varieties against Huh7it cells using the MTT assay, including cell viability, cytotoxicity, and IC_50_ data.
(XLSX)

S4 File Phytochemical and proximate composition data. Supporting data for the phytochemical profiling and proximate composition of pomegranate peel extracts, including LC-HRMS and GC-MS analyses and proximate composition measurements.
(XLSX)
