# Supplementary material for: Comparative phytochemical profiling and bioactivity evaluation of three Indonesian pomegranate (Punica granatum) peel varieties: Antioxidant, antibacterial, and in vitro antiproliferative activities
Source: PLoS One. 2026 Sep 15;21(9):e0357371. doi: 10.1371/journal.pone.0357371 (PMC13577413; doi:10.1371/journal.pone.0357371)
Supplement: Supplementary file 1 [file pone.0357371.s005.docx]

**Subject : Response to Reviewers**

**Manuscript ID:** PONE-D-26-19355

**Title:** *Comparative Phytochemical Profiling and Bioactivity Evaluation of Three Indonesian Pomegranate (Punica granatum) Peel Variety: Antioxidant, Antibacterial, and In vitro Antiproliferative Activities*

Dear Dr. Shengwei Sun,

We sincerely thank you and the reviewer for the careful evaluation of our manuscript and for providing constructive comments and valuable suggestions. We greatly appreciate the time and effort invested in reviewing our work.

We have carefully revised the manuscript according to all comments. The manuscript has been substantially improved in terms of scientific clarity, methodological description, statistical analysis, language quality, and overall organization. All modifications have been highlighted in the "Revised Manuscript with Track Changes", and detailed responses to each comment are provided in the attachment of this letter

We sincerely appreciate the reviewer's insightful comments, which have significantly improved the quality and clarity of our manuscript. We hope that the revised version satisfactorily addresses all concerns and will now be suitable for publication in PLOS ONE.

Thank you very much for your consideration.

Author,

Triono Bagus Saputro.

| No | Reviewer Comments | Response |
| --- | --- | --- |
| 1 | Editorial Requirement 1  Please ensure that your manuscript meets PLOS ONE's style requirements. | Thank you for this suggestion. The manuscript has been reformatted according to the latest PLOS ONE formatting guidelines, including manuscript structure, headings, references, tables, figures, and file naming conventions. |
| 2 | Editorial Requirement 2  Please provide details of the source of the pomegranates, including market location or company name and cultivar information. | We have revised the Materials and Methods section to clearly describe the origin of the pomegranate fruits, including the collection location, cultivar information, and sampling details.  (Page 4, Lines 143-149) |
| 3 | Editorial Requirement 3  Please provide a complete Data Availability Statement in the submission form, ensuring you include all necessary access information or a reason for why you are unable to make your data freely accessible. If your research concerns only data provided within your submission, please write "All data are in the manuscript and/or supporting information files" as your Data Availability Statement. | Thank you for this compliance directive. We have explicitly provided the Data Availability Statement in the online submission form as required. In accordance with the journal’s guidelines, since all the empirical data, quantitative findings, and analytical metrics generated in this study are fully disclosed within the text or its corresponding supplementary tables, our statement reads:  *"All data are in the manuscript and supporting information files."*  Furthermore, for the reader's convenience, this exact statement has also been maintained in the designated section at the end of the revised manuscript file (Page 27). |
| 4 | Editorial Requirement 4  We note you have included a table to which you do not refer in the text of your manuscript. Please ensure that you refer to Table 1, 2 and 3 in your text; if accepted, production will need this reference to link the reader to the Table. | Thank you for bringing this to our attention. We have thoroughly audited the entire text of the revised manuscript to ensure that all tables are explicitly and properly cited in the narrative before their respective appearances. We have verified that Table 1, Table 2, and Table 3 are now consistently and clearly referenced.  The primary in-text citations for each table are located at the following positions:   - Table 1 (LC-HRMS Metabolite Profiling) is explicitly cited in Line [721] inside the LC-HRMS results section. - Table 2 (GC-MS Phytochemical Profiling) is explicitly cited in Line [746] inside the GC-MS results section. - Table 3 Proximate analysis of purple pomegranate is explicitly cited in Line [767] inside the Proximate analysis results section. |
| 5 | Editorial Requirement 5  If the reviewer comments include a recommendation to cite specific previously published works, please review and evaluate these publications to determine whether they are relevant and should be cited. There is no requirement to cite these works unless the editor has indicated otherwise. | Thank you for this clarification. We have reviewed all comments provided by the reviewers and confirm that no requests or recommendations to cite specific previously published works were made. However, we have independently updated our reference list to ensure that all methodological adaptations are accurately and appropriately cited throughout the revised manuscript. |
| 6 | Comment to the Auhor 1  Is the manuscript technically sound, and do the data support the conclusions?  The manuscript must describe a technically sound piece of scientific research with data that supports the conclusions. Experiments must have been conducted rigorously, with appropriate controls, replication, and sample sizes. The conclusions must be drawn appropriately based on the data presented.  Reviewer #1: Partly | We acknowledge the reviewer’s initial evaluation regarding the technical rigor and data presentation of our manuscript.  To ensure our research framework is completely sound, fully reproducible, and rigorously supported by empirical data, we have executed a comprehensive overhaul across the entire manuscript. This includes standardizing concentration units and chemical nomenclature, clarifying IC50 mathematical models, and OD630 microbiological controls, verifying ANOVA parametric assumptions, expanding mass spectrometry identification criteria, and depositing all underlying datasets in S1–S4 Data.  Detailed point-by-point descriptions of these technical updates and structural revisions are provided in our responses to the specific General and Methods comments below. |
| 7 | Comment to the Auhor 2  Has the statistical analysis been performed appropriately and rigorously?  Reviewer #1: No | We acknowledge the reviewer’s concern regarding the statistical descriptions in our initial submission.  In the revised manuscript, we have completely overhauled the statistical framework to ensure full rigor, transparency, and reproducibility.  Detailed point-by-point descriptions of these statistical updates are provided in our responses to Comments #35 Below |
| 8 | Comment to the Auhor 3  The [PLOS Data policy](http://track.editorialmanager.com/CL0/http:%2F%2Fwww.plosone.org%2Fstatic%2Fpolicies.action%23sharing/1/010f019e5da389dd-b4143107-4e79-4e59-bb8b-9b272fd05c4a-000000/bh39PkJNbmFY3mmehGRHHkBCm5_nS2IhkI5zjHjJ75I=258) requires authors to make all data underlying the findings described in their manuscript fully available without restriction, with rare exception (please refer to the Data Availability Statement in the manuscript PDF file). The data should be provided as part of the manuscript or its supporting information, or deposited to a public repository. For example, in addition to summary statistics, the data points behind means, medians and variance measures should be available. If there are restrictions on publicly sharing data—e.g. participant privacy or use of data from a third party—those must be specified.  Reviewer #1: No | We acknowledge Reviewer’s initial concern regarding data availability. To ensure complete alignment with PLOS Data Policy, all raw, underlying, and individual quantitative datasets supporting the findings of this study have been deposited in full without restriction in the Supplementary Information files:   - S1 Data: Antioxidant Capacity (Raw absorbance, calibration curves, and IC50 calculations for TPC, TFC, DPPH, and ABTS). - S2 Data: Antibacterial Assay (Raw OD630 kinetics, blank corrections, and IC50 values). - S3 Data: Cytotoxicity Assay (Raw absorbance metrics and cell viability percentage datasets). - S4 Data: Phytochemical Profiling (Complete LC-HRMS and GC-MS raw annotations, peak area tables, library match scores, and retention metrics).   In addition, the Data Availability Statement in the submission form and manuscript text has been updated to read: *"All data are in the manuscript and/or supporting information files."* |
| 9 | Comment to the Auhor 4  PLOS ONE does not copyedit accepted manuscripts, so the language in submitted articles must be clear, correct, and unambiguous. Any typographical or grammatical errors should be corrected at revision, so please note any specific errors here.  Reviewer #1: No | We acknowledge Reviewer’s initial assessment regarding language and formatting.  The revised manuscript has undergone a thorough language and typographical editing process. All minor typos, formatting artifacts, and ambiguous phrasings have been meticulously corrected throughout the text. |
| 10 | Comment to the Auhor 5  Please use the space provided to explain your answers to the questions above. You may also include additional comments for the author, including concerns about dual publication, research ethics, or publication ethics. (Please upload your review as an attachment if it exceeds 20,000 characters) | We thank the Reviewer for the comprehensive feedback and suggestions. Please refer to our detailed, point-by-point response to the General Comments and specific line-by-line recommendations in Comment #11 onwards below. |
| 11 | Reviewer #1: General comments  The manuscript entitled “Comparative Phytochemical Profiling and Bioactivity Evaluation of Three Indonesian Pomegranate (Punica granatum) Peel Variety: Antioxidant, Antibacterial, and In vitro Antiproliferative Activities” addresses a relevant topic and may provide useful regional comparative data on Indonesian pomegranate varieties. However, the novelty should be presented more cautiously, as pomegranate peel is already well established as a source of antioxidant compounds. The manuscript would benefit from a clearer framing as a comparative phytochemical and bioactivity study, together with stronger support for LC-HRMS compound identification and a more appropriate statistical analysis. Provide means ± SD (or SE) and the number of biological and technical replicates for every quantitative result; include ANOVA/post hoc test details (assumptions checked, exact p values). Furthermore the Methods and Results alternately state that LC HRMS was performed on the “ethanol 96% extract” and that GC MS was performed on the “n hexane extract”, yet several bioassays and identification statements refer generically to “the extract” or to different solvents in different sections. For example: “The identification of bioactive compounds in the best performing antioxidant from the ethanol 96% extract was performed using Liquid Chromatography–High Resolution Mass Spectrometry (LC HRMS).” And later: “The chemical analysis of the best performing antioxidant from the n hexane extract of pomegranate peel was performed using GC MS.” Please harmonize the text so that each assay explicitly states which extract (solvent, tissue, and variety) was used and why that extract was selected for that analysis. The manuscript appears to analyse only the “best performing” extract in some downstream assays (LC HRMS, GC MS, proximate analysis) but does not justify the selection criteria or indicate whether representative replicates were profiled. State the objective criteria used to choose samples for chemical profiling and whether profiling was performed on pooled or individual replicates. For the reasons outlined above, in my opinion the Materials and Methods section requires substantial revision for clarity, accuracy, and reproducibility. Moreover, the Results section should be largely rewritten, as the findings are currently not presented in a clear and well-structured manner. Several paragraphs include generic background descriptions rather than direct results, and the quantitative outcomes are not always linked to appropriate statistical support. The authors should revise this section to present only the experimental findings, organize them logically, and clearly distinguish data interpretation from general discussion.  The Discussion section requires substantial rewriting. In its current form, it is largely descriptive and repetitive, and several paragraphs read as background information or a repetition of the Results rather than a critical interpretation of the findings. The authors should reorganize the Discussion around the main experimental outcomes: antioxidant capacity, antibacterial activity, antiproliferative activity, and phytochemical profiling. Each point should be interpreted in relation to the statistical results and compared critically with the literature. Overall, the Discussion should be extensively rewritten to improve clarity, coherence, and scientific rigor. The authors should remove generic background statements, avoid repeating the Results, resolve internal contradictions, provide statistical support for reported differences, and interpret the findings more cautiously. | We sincerely thank Reviewer #1 for this comprehensive and constructive evaluation. In accordance with your guidance, we have executed a major overhaul of the entire manuscript to ensure technical soundness, mathematical accuracy, and full reproducibility:   1. Reframed Focus & Matrix Transparency: The Introduction and workflow were reframed to present a cautious comparative study across separated peel tissues (exocarp vs. mesocarp) of three Indonesian cultivars. Generic terms were eliminated, and explicit solvent-tissue-cultivar designations were applied throughout. Clear selection criteria for downstream profiling (LC-HRMS, GC-MS, proximate) were detailed. 2. Overhauled Methodology & IC50 Precision: Units were standardized µgmL^-1^ water terminology corrected ("distilled water"), and vehicle/matrix blanks incorporated. Mathematical models for IC50 were explicitly separated—linear for n-hexane (IC50 = (50-a)/b) and exponential for ethanolic extracts (IC50 = e^{(50-a)/b})—backed by validated references (Sukweenadi et al.; Elmezwghy et al.). Microbiological protocols (OD630 microplate reader, Shinrinken Lab) and MS identification criteria (tentative assignments, contaminant isolation) were fully detailed. 3. Rigorous Statistics: Specified three independent biological replicates (in technical triplicate), verified ANOVA parametric assumptions (Shapiro-Wilk and Levene's tests), incorporated exact p-values (p < 0.05), and justified Duncan's test (DMRT) for cultivar differentiation. 4. Restructured Results & Discussion: The Results section was sanitized to contain strictly empirical data and statistical outputs, removing all textbook background. contain strictly empirical data and statistical outputs. The Discussion section was extensively rewritten into dedicated thematic sections to address all internal contradictions and align with statistical findings:   Contradictions Resolved: Clarified that high TPC/antioxidant capacity in the exocarp applies to ethanolic extracts, whereas the mesocarp showed higher activity in n-hexane extracts.  Separation of Chemical Profiles: Separated the discussion of ethanolic purple exocarp (LC-HRMS) from n-hexane red mesocarp (GC-MS) to prevent conflation, adding critical discussions on non-phenolic features and identification confidence.  Statistical & Literature Alignment: Accompanied all comparative statements with explicit p-values.  Detailed point-by-point responses to all specific line comments are provided in the response to comments bellow. |
| 12 | L60–62:  The statement comparing the present results with Andriani’s study is not sufficiently supported. The manuscript compares “purple pomegranate” with “black pomegranate” without clarifying whether these terms refer to the same cultivar/color category or to distinct genotypes. This is relevant because antioxidant activity in pomegranate peel is strongly influenced by cultivar identity and phytochemical composition, not simply by peel colour. Without clarification of cultivar equivalence and methodological comparability, the reported discrepancy with Andriani’s findings remains descriptive and may be misleading. | We completely agree with the reviewer’s constructive critique regarding the potential ambiguity of comparing the "purple pomegranate" in this study with the "black pomegranate" from Andriani's research without definitive genotypic clarification.  We acknowledge that antioxidant profiles in pomegranate peels are highly dependent on specific cultivar identities, environmental factors, and precise extraction methodologies, rather than visual peel color alone.  To prevent any misleading interpretations or unsupported assumptions regarding cultivar equivalence, we have completely removed the statement comparing our results with Andriani's study from the manuscript. The section has been thoroughly revised to focus strictly on the established gaps in literature regarding Indonesian pomegranate cultivars, ensuring the text remains rigorous and objectively supported by our direct experimental data. |
| 13 | L63–81:  The study objective should be stated more clearly and concisely. The final paragraph of the Introduction includes excessive methodological detail and should be shortened, leaving only the rationale and the specific aim of the work. | We sincerely appreciate the reviewer’s valuable guidance on sharpening the focus of our study's objective. In accordance with your suggestion, we have thoroughly restructured and shortened the final paragraphs of the Introduction. We have removed the excessive methodological descriptions and focused strictly on the critical research gaps (the rationale) and the clear, specific aims of this work.  The revised section now explicitly highlights the lack of comprehensive data on separated pomegranate peel tissues (exocarp vs. mesocarp) across Indonesian cultivars using contrasting solvent polarities.  (Lines [111-118]): |
| 14 | L90:  The drying protocol for pomegranate peel should be described in greater detail. The authors should report the drying conditions, including ambient temperature, if available. | Thank you for pointing out this omission. We have now fully updated the Materials and Methods section to include the specific and precise operating parameters for the drying process, including the exact temperature, duration, and equipment used, ensuring full reproducibility of the sample preparation.  Please refer to the updated text in the manuscript (Lines [138-145]). |
| 15 | L113:  This sentence should be moved to the Results section. | We sincerely thank the reviewer for highlighting the misplacement of this sentence. Upon careful re-evaluation of the manuscript structure and the validity of the statement, we recognize that this generalization regarding extraction yield and bioactive content is scientifically oversimplified. A higher crude extract yield does not necessarily correlate with a higher concentration of target bioactive metabolites, as the mass may be heavily influenced by non-target structural ballasts (e.g., primary carbohydrates or bulk lipids).  Furthermore, since the primary objective of this study focuses on the comparative profiling of standardized phytochemical concentrations (TPC, TFC) and specific bioactivities rather than raw extraction efficiencies, we have decided to completely remove this sentence from the manuscript. This removal ensures that the text remains scientifically rigorous and avoids speculative claims without presenting the complete raw extraction yield datasets. |
| 16 | L116:  The authors should use an appropriate term, such as “distilled water” or “deionized water”, and rephrase the sentence. For example: “A stock solution of the crude extract was prepared…” | We thank the reviewer for this precise and helpful suggestion to improve the clarity and professional tone of the manuscript. In accordance with your guidance, we have corrected the terminology throughout the manuscript to "distilled water" and "deionized water" where appropriate.  The updated sentence now reads as follows (Lines [139, 166, 205, 220, 234, 239, 245, 268, 279, 287, 321, 322, 338]): |
| 17 | L120–132:  The calibration procedure is unclear. The concentrations reported as 100–3.125 µg mL⁻¹ appear to refer to the working gallic acid standards, not to the final reaction mixture. Since 0.20 mL of standard solution is diluted to a final volume of 4.20 mL, the final assay concentrations are 21-fold lower. The authors should clarify which concentrations were used to construct the calibration curve and how dilution factors were applied in the GAE calculation. | We are highly grateful to the reviewer for this rigorous and insightful comment regarding the dilution configurations within the colorimetric reaction mixtures.   1. Calibration Scale & Mathematical Validity: We clarify that the calibration curves for both Total Phenolic Content (TPC) and Total Flavonoid Content (TFC) were constructed using the concentrations of the initial working standard solutions (3.125–100 μg/mL) rather than the final reaction volumes. To ensure mathematical consistency and avoid manual dilution biases, the pomegranate exocarp and mesocarp extracts were introduced and analyzed using the exact same volumes and configurations (0.2 mL of sample for TPC into a final volume of 4.2 mL; 1.0 mL of sample for TFC into a final volume of 7.0 mL). Because these respective dilution factors (21-fold for TPC and 7-fold for TFC) applied identically to both the chemical standards and the unknown sample extracts, the dilution factors mathematically cancel out when back-calculating concentrations using the regression equations. We have explicitly stated this in the text and introduced the formal equations used to calculate GAE and QE. 2. Dual-Solvent Rationale: We have explained that distilled water was utilized for the ethanol extract standards, while dimethyl sulfoxide (DMSO) was utilized for the n-hexane extract standards. This setup was strictly chosen to optimize solubility and ensure matrix compatibility, as gallic acid and quercetin exhibit limited solubility in non-polar environments like n-hexane. 3. Solvent Blanks: We have updated the text to explicitly state that corresponding solvent blanks (distilled water and DMSO subjected to the identical chemical reagents) were prepared and run concurrently as baseline references to zero the spectrophotometer, thereby eliminating any potential background absorbance from the solvent matrices.   The manuscript has been thoroughly updated in both the TPC sub-sections (Lines [204-228]) and TFC sub-sections (Lines [231-258]) to reflect these transparent parameters and clarify the use of technical triplicates to ensure instrumental precision. |
| 18 | L144–148: See the comment above. | In alignment with our response to the previous comment regarding the TPC assay, we have applied the exact same rigorous methodological updates and formatting corrections to the Total Flavonoid Content (TFC) protocol in this section:   1. Standardization of Concentration Units: All concentration metrics in the TFC section previously written as "ppm" have been systematically updated to the standard unit µgmL^-1^ 2. Correction of Water Terminology: The term "aquabides" has been completely replaced with "distilled water" to comply with standardized chemical nomenclature. 3. Clarification of Solvent Preparation and Blanks: We have clarified that the standard Quercetin and extract working solutions were derived from DMSO stock solutions and subsequently diluted in distilled water. To eliminate any potential background absorbance interference from the solvent matrix, appropriate corresponding solvent blanks (DMSO-distilled water vehicle without the active compounds) were analyzed concurrently and utilized for baseline subtraction.   The fully updated and standardized TFC methodology can be found in Lines [230-258] of the revised manuscript. |
| 19 | L155–156: Please check the sentence “standard curve of gallic acid”, as this section refers to quercetin standards for TFC determination. | We sincerely apologize for this typographical oversight. We have thoroughly reviewed the section and corrected the phrasing from "gallic acid" to "quercetin" to accurately reflect the reference standard utilized for the Total Flavonoid Content (TFC) calibration and quantification.  The corrected sentence now accurately states the use of the quercetin standard curve and has been updated in the manuscript (Lines [248]) |
| 20 | L172–178:  The method should be reported more clearly, using standard concentration units, preferably µg mL⁻¹ rather than ppm. The term “aquabides” should be replaced with double-distilled or deionized water. The rationale for preparing standards in both water and DMSO should also be explained, and appropriate solvent blanks should be included. | We sincerely thank the reviewer for these highly constructive methodological recommendations. We have thoroughly revised the designated section to enhance clarity and scientific accuracy as follows:   1. Standardization of Concentration Units: All concentration metrics previously expressed in "ppm" have been systematically converted to the standard unit µg/mL throughout the manuscript. 2. Correction of Water Terminology: The localized term "aquabides" has been completely removed and replaced with "distilled water" to comply with international chemical nomenclature. 3. Rationale for Standards in Water and DMSO: We have clarified the preparation protocol in the text. Reference standards were prepared using DMSO as the primary stock solvent to ensure complete solubilization of highly lipophilic or moderately polar phenolic/flavonoid structures, which then underwent subsequent dilutions in distilled water to match the exact matrix of the working assay conditions. 4. Inclusion of Solvent Blanks: To eliminate any spectrophotometric background interference caused by the organic modifier, appropriate solvent blanks containing the exact corresponding ratios of DMSO and distilled water (without the active extracts/standards) were concurrently analyzed and utilized for baseline subtraction.   The corrected and expanded methodology can be found in Lines [230-258] of the revised manuscript. |
| 21 | L199–214:  The introductory description of the ABTS method should be revised or removed from the Materials and Methods section. The statement that ABTS is “also known as ABTS/TAC or ABTS/TEAC” is inaccurate: ABTS is the reagent/assay system, whereas TEAC refers to Trolox equivalent antioxidant capacity, and TAC is a broader analytical concept. In addition, ABTS itself is not “produced” by oxidation; rather, the ABTS radical cation, ABTS•⁺, is generated by reacting ABTS with potassium persulfate. Since the authors subsequently describe the actual reagent preparation, this paragraph should focus directly on the experimental protocol and avoid general textbook-style statements. Moreover, there is an inconsistency between the use of a Trolox standard curve and the expression of results as IC₅₀. If results are expressed as IC₅₀, the authors must test a concentration series of each extract and calculate the concentration producing 50% inhibition. Conversely, if a Trolox calibration curve is used, results should be expressed as Trolox equivalents. The current description mixes the two approaches and should be clarified. | We sincerely thank the reviewer for this rigorous, highly accurate, and constructive critique of our ABTS methodology description. We acknowledge the conceptual and terminological errors in our initial draft regarding the distinction between ABTS, TEAC, and TAC, as well as the incorrect phrasing regarding the generation of the radical cation. Furthermore, we appreciate the warning regarding the methodological inconsistency between the description of a Trolox calibration curve and the final presentation of IC50 values.  To address all the concerns raised, we have completely overhauled the Materials and Methods section as follows:   1. Removal of Textbook-Style Introduction and Correction of Chemistry: The introductory paragraph (formerly Lines 199–214) has been completely removed. We have eliminated the inaccurate references to "TAC/TEAC" and corrected the chemical narrative. The section now focuses directly on the experimental protocol, accurately describing that the active ABTS radical cation was generated through the controlled reaction of ABTS with potassium persulfate. 2. Clarification of Trolox Function: We have corrected the mixed analytical approaches. Trolox was employed strictly as a positive control to validate the baseline performance and stability of the radical system, and *not* as a calibration curve for quantifying the samples into Trolox equivalents. 3. Explicit Specification of Sample Concentration Series: To align perfectly with the empirical IC50 results presented in the manuscript, we have explicitly detailed the separate concentration series tested for each solvent matrix (125, 250, 500, and 1000µg mL^-1^ for the non-polar n-hexane extracts; 31.25, 62.5, 125, 250, and 500 µg mL^-1^ for the polar 96% ethanolic extracts). 4. Integration of Solvent-Specific Mathematical Models: We have explicitly stated the two distinct mathematical frameworks used to calculate the final IC_50_ metrics. The n-hexane extracts were mathematically resolved using a standard linear regression curve IC_50_ = (50 - a)/b) according to the protocol by Sukweenadi et al., whereas the ethanolic extracts were resolved using an exponential transformation of a natural logarithm regression model (IC_50_ = e^(50-a)/b) following the method optimized by Elmezwghy et al.   The fully revised, protocol-focused text can be found in Lines [312-360] of the revised manuscript, ensuring full transparency and methodological consistency. |
| 22 | L232:  The authors should clarify and correct the description of the IC₅₀ calculation. As written, the formula IC₅₀ = (50 − a) / b is only valid when the linear regression relates percentage inhibition directly to concentration. If the regression was performed using ln(concentration) as the predictor, the correct expression is IC₅₀ = exp[(50 − a) / b]. The authors should explicitly state which predictor was used and apply the corresponding formula. | We sincerely thank the reviewer for identifying this mathematical oversight. We completely agree that applying the standard linear formula IC50 = (50 - a)/b to a natural logarithm (ln) transformed dataset is mathematically incorrect and would lead to erroneous evaluations.  In accordance with your critical guidance, we have thoroughly corrected and expanded the description of the IC0 calculations in the Materials and Methods section (for both the DPPH and ABTS assays). We have explicitly stated the specific predictors used for each extraction matrix and applied the exact corresponding mathematical formulas:   1. For the non-polar *n*-hexane extracts: The linear regression was performed using the original concentration values as the direct predictor (y = bx + a), following the protocol by Sukweenadi et al. Consequently, the IC50 was calculated using the conventional linear equation:   IC_50_ = (50 – a)/b   2. For the polar 96% ethanolic extracts: The linear regression was performed using the natural logarithm of the concentration [ln(concentration)] following the method by Elmezwghy et al. To mathematically resolve the true concentration value, the inverse function (exponential) was applied. The formula has been corrected to:   $IC50= e^{(\frac{50-a}{b})}$  The text in the manuscript has been fully updated to display these two distinct equations alongside their respective biological solvent rationales and reference citations (Lines [297-310] and Lines [346-360]). |
| 23 | L241:  Please correct the typo “ThermoScientific™ Accucore™” to “Thermo Scientific™ Accucore™”, and check the manuscript for inconsistent brand formatting. | We thank the reviewer for their meticulous observation. We have corrected the typographical error from "ThermoScientific™ Accucore™" to "Thermo Scientific™ Accucore™" in the revised manuscript (Line [371]).  Additionally, in accordance with your recommendation, we have thoroughly audited the entire manuscript to ensure that all manufacturer names and brand formatting (such as Merck, Sigma-Aldrich, Thermo Scientific, etc.) are standardized, chemically accurate, and completely consistent throughout the text. |
| 24 | L243–245:  The elution program is ambiguous. The authors should clarify it by providing explicit time points and hold durations, including initial conditions, gradient endpoint, hold period, return to initial conditions, and re-equilibration time. | We thank the reviewer for requesting this essential chromatographic detail. We have completely revised the description of the mobile phase gradient to provide full transparency regarding the exact timeline, hold durations, and re-equilibration steps.  These detailed gradient parameters can now be found in Lines [373-377] of the revised manuscript. |
| 25 | L250:  A single spray voltage is reported, although acquisition was performed in both positive and negative ion modes. The authors should specify the spray voltage used for each polarity and describe the polarity-switching strategy. | We appreciate the reviewer's precise technical evaluation regarding the mass spectrometry source configuration. In our analytical setup, mass spectrometric detection was executed using an automated polarity-switching strategy integrated within the Q Exactive Orbitrap system, enabling the sequential acquisition of full MS and data-dependent MS^2 (dd-MS^2) spectra for both positive and negative ionization networks within a single chromatographic injection.  Regarding the spray voltage, a standardized voltage of 3.30 kV was configured and automatically maintained by the instrument control software across both positive and negative polarity cycles, which provided optimized and reproducible ionization efficiencies for the target multi-class phenolics and organic compounds under the current mobile phase composition. We have clarified this operational layout in the text (Line [378-384]). |
| 26 | LC-HRMS identification criteria: The authors should specify the compound identification criteria, including mass tolerance, MS/MS matching thresholds, databases or libraries used, and whether authentic standards were employed for confirmation. | We completely agree that defining the exact parameters for data processing is critical for validating high-resolution mass spectrometry annotations. We have expanded the description of our data processing workflow:   1. Software and Libraries Used: Raw data processing and untargeted metabolite annotation were executed using Thermo Scientific™ Compound Discoverer™ version 3.2, channeling automated structural queries against the ChemSpider and mzCloud spectral libraries. 2. Identification Criteria: Compounds were annotated using strict strict filters including accurate mass measurements (Δmass ≤ 5 ppm tolerance threshold), predicted molecular formula generation, retention time mapping, database matching scores, and automated MS/MS fragmentation matching generated within the *Compound Discoverer*. 3. Confirmation Status: As authentic reference standards were not utilized for validation, we have explicitly stated in the text that all identified features are classified as tentative annotations based on high-resolution mass spectrometric guidelines.   The updated methodology detailing these criteria is available in Lines [385-395] of the revised manuscript. |
| 27 | L256:  Why was only the n-hexane extract selected for GC-MS analysis? The selection criterion should be clearly justified. | We thank the reviewer for highlighting the need to justify our solvent selection workflow. GC-MS is fundamentally optimized for the separation and detection of volatile, thermally stable, and non-polar to semi-polar low-molecular-weight compounds.  Because n-hexane serves as a highly specialized non-polar vehicle, it was exclusively selected to capture the lipophilic fractions, essential fatty acids/esters, and volatile hydrocarbon components of the pomegranate mesocarp matrix that would otherwise be poorly resolved or lost in high-polarity aquatic or ethanolic extraction streams.  Conversely, our primary screening confirmed that the polar fractions (96% ethanolic extract) possessed the vast majority of target bioactive molecules (phenolics and flavonoids), which were appropriately routed into the LC-HRMS platform designed for highly polar compounds. We have integrated this strategic rationale into the text to avoid any ambiguity (Lines [192-194]). |
| 28 | L267:  Were authentic standards used to confirm compound identification? If not, the level of identification confidence should be clearly stated. | We acknowledge the reviewer's concern regarding identification confidence levels. No authentic reference chemical standards were employed for compound verification in this study.  To maintain strict adherence to scientific reporting standards, we have explicitly updated both the LC-HRMS and GC-MS text sections to clearly report that all compound identities presented in this work must be regarded strictly as tentative annotations based on structural comparisons with the NIST mass spectral library, ChemSpider, and mzCloud databases.  The text has been updated to reflect this limitation in Lines [393 and 415] of the revised manuscript. |
| 29 | L277:  The authors should provide additional information on the origin of the bacterial strains. | We thank the reviewer for pointing out this omission. We have updated the Materials and Methods section to explicitly state the specific biological source and repository of the test microorganisms.  The text now specifies that the *Escherichia coli* and *Staphylococcus aureus* strains were obtained from the official bacterial culture collection of Department of Chemistry, Faculty of Science and Data Analytics, Institut Teknologi Sepuluh Nopember.  (Lines [434]). |
| 30 | L279:  The sentence “was examined for the pomegranate peel ethanolic extract” should be revised.  More importantly, the rationale for using ethanolic extracts in some assays and n-hexane extracts in others is unclear. In my opinion, the Materials and Methods section requires careful revision because several inconsistencies are present. | We thank the reviewer for highlighting this phrasing ambiguity and requesting clarification on our solvent screening workflow.   1. Revision of Terminology: The ambiguous sentence has been completely rephrased to ensure specific matrix nomenclature 2. Scientific Rationale for Solvent Selection: We have clarified the experimental design in the text. *n*-Hexane was utilized strictly to isolate the non-polar/lipophilic fractions, which were subsequently analyzed via GC-MS to identify volatile constituents. However, our preliminary screening of phytochemicals (TPC and TFC) and antioxidant parameters (DPPH and ABTS) clearly demonstrated that the *n*-hexane fractions possessed extremely low bioactive components and negligible antioxidant performance compared to the polar fractions. 3. Because antimicrobial compounds in plant matrices are well-documented to be predominantly polar phenolics, flavonoids, and glycosides, only the 96% ethanolic extracts (which exhibited superior TPC, TFC, and radical scavenging baselines) were selectively channeled into downstream biological assays (antibacterial and MTT assays). This targeted workflow ensures that biological testing was focused on the most active, high-yielding fraction to maximize resource efficiency. The section has been carefully revised to reflect this logical flow. |
| 31 | L289–293:  OD measurements are reported at 630 nm and converted to CFU estimates. The bacterial inoculum appears very high, since OD₆₃₀ = 0.4 is reported to correspond to approximately 10⁸–10⁹ CFU mL⁻¹ before dilution. The authors should provide the basis for these conversions, such as a calibration curve or McFarland standard, and either use the more conventional OD₆₀₀ or explain why OD₆₃₀ was chosen. The actual CFU mL⁻¹ used in the assay and the number of biological replicates should also be reported. | We highly appreciate the reviewer’s rigorous evaluation of our microbiological parameters and density benchmarks:   1. Basis for OD to CFU Conversion: The correlation between OD630 = 0.4 and the respective colony-forming units 10⁹ CFUmL−1 for *Escherichia coli* and 10⁸ CFUmL−1 for *Staphylococcus aureus* was established using a pre-calibrated colony-count regression curve (serial dilution and subsequent agar plate counting) established by the Shinrinken Laboratory, Kyushu University, from which this automated protocol was adopted. 2. Rationale for Selecting 630 nm over 600 nm: While 600 nm is standard for visual microdilution, 630 nm was intentionally chosen because it represents the fixed operational filter integrated into the microplate reader platform used for this automated high-throughput assay. Wavelengths between 620–650 nm are widely recognized and validated in standard protocol manuals (including automated systems) for bacterial turbidimetric measurements established by the Shinrinken Laboratory, Kyushu University, from which this automated protocol was adopted. 3. Actual CFU Inoculum and Replicates: We have clarified in the text that the bacterial suspension was further diluted in the final assay mixture to reach an operational cell density of approximately 10^6–10^7 CFU mL^-1^ per well, preventing over-saturation. All antibacterial assays were conducted in three independent biological replicates, each consisting of technical triplicates, to ensure statistical validity.   The methodology detailing these criteria is available in Lines [429-480] of the revised manuscript. |
| 32 | L298–306:  The authors should explain how the final extract concentrations were achieved in the wells, including stock concentration, dilution scheme, and final assay volume. The concentration range tested and the final solvent concentration in the wells should be reported. Although the authors state that DMSO did not exceed 1% v/v, the final well concentration should be explicitly confirmed. The concentration of the positive control, sorbic acid, should also be specified, and the rationale for its selection should be provided. | We thank the reviewer for requesting these operational details, which are critical for assay reproducibility. We have expanded the description in the revised manuscript:  Dilution Scheme and Concentrations: The dried extracts were first dissolved in pure DMSO to prepare a stock concentration of 20 mg mL^-1^. To establish dose-response curves for IC50 determination, serial dilutions of the pomegranate ethanolic extracts were prepared to yield final test concentration ranges (2000, 1000, and 500 µg mL−1).  These parameters have been explicitly detailed in Lines [453-455] of the revised manuscript. |
| 33 | Sterility controls, vehicle controls, and extract blanks should be clearly described. Since plant extracts may cause turbidity or absorbance at the measurement wavelength, the authors should state how blank absorbance was handled to correct for extract colour or turbidity. Additional verification, such as viable counts or an alternative viability assay, would strengthen the reliability of the OD-based measurements. | We thank the reviewer for requesting these operational details, which are critical for assay reproducibility. We have expanded the description in the revised manuscript   1. Clear Description of Control Configurations:    - Sterility Controls: Uninoculated Nutrient Broth (NB) without extracts or bacteria to verify media purity.    - Vehicle Controls (Negative Control): NB + bacterial inoculum + 1% (v/v) DMSO (without active extract) to establish baseline maximum bacterial growth.    - Extract Blanks (Matrix Background Correction): Dedicated wells containing NB + respective extract concentrations without bacterial inoculum were run concurrently in parallel. 2. Handling of Extract Color and Turbidity Artifacts:   To eliminate interference caused by the natural intense coloration and variable turbidity of pomegranate peel extracts, raw OD630 readings were systematically corrected before calculating growth inhibition percentages. The net absorbance (A sample) was mathematically resolved using the equation:  $\left( A_{sample}=A_{measured}-A_{blank} \right)$  where $A_{measured}$ is the absorbance of the extract-treated bacterial culture, and $A_{blank}$ is the absorbance of the corresponding uninoculated extract blank).   3. Inoculum Verification:   We have clarified in the revised text that the bacterial density (OD630 = 0.4) and the operational cell density used in the microplate assay (approx. 10^6^-10^7^ CFU mL^-1^) were verified via serial dilution and viable colony counts on Nutrient Agar plates according to the validated Shinrinken Laboratory protocol (Kyushu University).  The revised paragraph reflecting these rigorous control and background correction procedures can be found in Lines [458-482] of the revised manuscript. |
| 34 | L322:  Please correct the typo “cacer cells”. | Thank you for bringing this typographical error to our attention. We have corrected the typo from "cacer cells" to "cancer cells" in the revised manuscript (Line [498]). We have also double-checked the surrounding text to ensure all spelling and technical terms are accurate. |
| 35 | L339–341:  The statistical analysis is insufficiently described. The authors should clearly specify the variables analysed, the experimental unit, the number and type of replicates, the significance threshold, and whether ANOVA assumptions were checked. The use of Duncan’s test should also be justified, or replaced with a more appropriate multiple-comparison procedure. As currently written, the statistical approach is too generic and not fully reproducible. | We sincerely thank the reviewer for this rigorous and constructive critique. We agree that the initial statistical description was too generic. We have thoroughly revised the Statistical Analysis section to provide absolute transparency and ensure full reproducibility. The specific updates include:   1. Specification of Replicates and Experimental Units: We have explicitly stated that all assays were performed in three technical triplicate. 2. Variables and Stratification Strategy: We have clarified the analytical framework. The independent variables analyzed were the pomegranate cultivars (red, yellow, purple), while the dependent variables were TPC, TFC, IC50 values of ABTS, DPPH, and antibacterial activity, and cytotoxicity percentages. To avoid multi-factor interaction artifacts, data were stratified and analyzed independently based on the tissue type (exocarp vs. mesocarp) and solvent system (ethanol vs. *n*-hexane). 3. Verification of ANOVA Assumptions: In accordance with your guidance, we have explicitly confirmed in the text that data normality and homogeneity of variances were verified using Shapiro-Wilk and Levene’s tests, respectively, ensuring all datasets strictly satisfied the mathematical assumptions required for parametric One-Way ANOVA. 4. Justification of Duncan’s Multiple Range Test (DMRT): We have added a scientific rationale for selecting DMRT as our post-hoc procedure. DMRT was chosen because our datasets demonstrated strict homogeneity of variance, and this specific test provides high statistical power and sensitivity required to resolve subtle, yet biologically significant, inter-cultivar variations among the three Indonesian pomegranate lines, which might be overly masked by conservative post-hoc tests like Tukey.   The fully detailed, expanded, and reproducible Statistical Analysis section is now available in Lines [515-525] of the revised manuscript. |
| 36 | L358–369:  This section reads as background information rather than experimental results. Unless the authors performed anatomical or histological analyses, the description of exocarp and mesocarp tissues is unnecessary in the Results section and should be removed or relocated. The Results section should focus on the data generated in this study. | We completely agree with the reviewer’s perspective. Since this study did not involve direct anatomical, histological, or microscopic evaluations of the pomegranate fruit tissues, including general descriptive background of the exocarp and mesocarp within the Results section was structurally inappropriate.  To ensure that the Results section remains strictly data-driven and focused exclusively on the empirical findings generated in this study, we have completely removed the descriptive text regarding the tissue anatomy from the manuscript. The paragraph has been deleted (formerly Lines 358–369), ensuring the manuscript structure aligns with standard scientific reporting guidelines. |
| 37 | L382–383:  Pearson correlation analysis is mentioned in the Results, but it is not described in the Materials and Methods section. The authors should either describe this analysis in the statistical methods or remove the statement. | We completely agree with the reviewer’s comment regarding the inconsistency of mentioning the Pearson correlation analysis without defining it within our methodology.  Upon re-evaluating the manuscript's statistical framework, we determined that our primary conclusions are already robustly supported by the comparative analysis of variance (ANOVA) and the explicit regression models used for IC50 derivation. Therefore, to streamline the findings and ensure absolute consistency between the methodology and the results, we have completely removed the statement regarding the Pearson correlation analysis from the manuscript. |
| 38 | L388–389:  When reporting significant effects, the authors should provide the corresponding p-values. This should be checked and revised throughout the manuscript. | We thank the reviewer for this crucial statistical recommendation. We fully agree that reporting exact or threshold-based p-values is essential for quantitative transparency and rigor.  In accordance with your guidance, we have thoroughly audited the entire manuscript—with special attention to the Total Phenolic Content (TPC) results section (Lines 388–389)—to ensure that every claim of statistical significance is explicitly accompanied by its corresponding p-value descriptor. All relevant data sentences and graph captions have been updated to explicitly state the significance thresholds (e.g., p < 0.05) derived from our One-Way ANOVA and post-hoc analyses.  The updated statistical reporting for the TPC section can be found in Lines [558-579] of the revised manuscript. |
| 39 | L442 and L448:T  These interpretative comments should be moved to the Discussion section. | We completely agree with the reviewer’s structural assessment. In the original draft, several interpretative statements and theoretical explanations regarding the DPPH radical scavenging mechanism and cultivar dependencies were incorrectly placed within the Results section.  To maintain a strict separation between factual data reporting and scientific interpretation, we have thoroughly revised the DPPH results subsection. All speculative or comparative discussion remarks have been completely extracted from the Results section, leaving only the objective empirical data presentations (IC_50_ values, standard deviations, and ANOVA metrics). Concurrently, the removed interpretative comments have been successfully relocated and integrated into the global Discussion section.  The streamlined DPPH results text can be found in Lines [603-629]. |
| 40 | L460:  Please correct the typo “$”. | We apologize for this typographical error. The stray character “$” has been completely removed from the text, and the sentence has been corrected in the revised manuscript (Line [633]). We have also reviewed the document to ensure no other formatting artifacts remain. |
| 41 | L462–465:  This sentence should be checked and revised for clarity. | We thank the reviewer for highlighting the ambiguity and awkward phrasing in these sentences. We have completely restructured and rewritten this paragraph in the revised manuscript to ensure maximum narrative clarity, precise matrix nomenclature, and consistent reporting of statistical significance alongside standard deviations. The updated, clear, and statistically validated text can be found in Lines [637-643] of the revised manuscript. |
| 42 | L467:  The sentence “Overall, the ethanolic peel extracts demonstrated stronger activity than their mesocarp counterparts” should be checked and revised. The authors may mean that ethanolic exocarp extracts showed stronger activity than ethanolic mesocarp extracts, but this needs to be clearly stated. The paragraph should be carefully revised to distinguish between exocarp and mesocarp extracts. | We thank the reviewer for identifying this ambiguous phrasing and highlighting the need to strictly distinguish between exocarp and mesocarp tissue layers in our comparisons.  We have thoroughly revised the paragraph to ensure precise matrix nomenclature throughout the text, clearly differentiating between solvent types (ethanol vs. n-hexane) and tissue layers (exocarp vs. mesocarp). The concluding summary sentence of this subsection has been updated to explicitly reflect these distinctions: |
| 43 | L482–483:  Please clarify this point. Why did the authors not determine the minimum inhibitory concentration (MIC)? MIC is one of the most widely used parameters for assessing antimicrobial activity and is generally more informative from an applicative perspective, as it indicates the concentration required to inhibit visible bacterial growth. Another relevant parameter is the minimum bactericidal concentration (MBC). Did the authors evaluate MBC as well? | We sincerely thank the reviewer for this important methodological question regarding standard microbiological parameters. We acknowledge that MIC (visual determination of growth inhibition) and MBC (minimum bactericidal concentration via agar plating) are the traditional benchmark parameters in qualitative antimicrobial screenings.  However, in this study, the antibacterial evaluation was conducted using a quantitative spectrophotometric approach OD630 microplate reader measurement) rather than visual turbidity assessment. This approach was strictly adapted from the standardized protocol of the Shinrinken Laboratory, Kyushu University.  The rationale for selecting the half-maximal inhibitory concentration IC50 over the conventional MIC/MBC in this specific experimental design includes:   1. Precision and Quantifiability: Measuring optical density at 630 nm allows for the precise quantification of minor shifts in bacterial cell density. Unlike visual MIC, which relies on subjective human observation and provides a single discrete concentration step, the OD-based approach generates continuous quantitative data to construct comprehensive dose-response curves. 2. Mathematical Accuracy via Kinetical Curve Fitting: The bacterial IC50 represents a mathematically resolved parameter that defines the exact concentration required to suppress 50% of the active bacterial population growth relative to the untreated control. This metric is highly sensitive and widely accepted in automated high-throughput drug screening platforms. 3. Absorbance Background Correction: Pomegranate extracts possess an intense natural color and variable turbidity, which can severely mask visual interpretation in standard MIC assays. Utilizing the spectrophotometric microplate reader protocol allowed us to deploy dedicated blank wells (nutrient broth + extract without bacteria) to meticulously subtract background absorbance interference, ensuring the resulting IC50 curves are mathematically valid and free from matrix artifacts.   Since MBC determinations require secondary sub-culturing onto solid agar plates from visual MIC endpoints—which were not part of the automated OD-based kinetic protocol developed by the Shinrinken Laboratory—MBC values were not evaluated in this study. To prevent any confusion for future readers, we have ensured that the terminology in the Materials and Methods section explicitly defines the automated spectrophotometric nature of the protocol and states that the antibacterial efficiency is strictly quantified and expressed as mathematical IC50 values. |
| 44 | L488-491:  In this section, the authors should report only the results. Interpretation and discussion of the findings should be moved to the Discussion section. | We completely agree with the reviewer’s recommendation regarding the structural separation between the Results and Discussion sections.  In accordance with your guidance, we have thoroughly audited the antibacterial results subsection. All interpretive remarks, speculative explanations of the mechanism of action, and comparative discussions against prior literature have been meticulously extracted from this section. The text in the Results section has been streamlined to report exclusively the empirical data, standard deviations, and statistical outputs generated from our assays. Concurrently, all the removed interpretative and discussion elements have been appropriately relocated and integrated into the global Discussion section of the manuscript.  The streamlined, antibacterial results can now be found in Lines [659-693]. |
| 45 | L507:  “The antiproliferative efficiency of pomegranate peel against cancer cells was examined for the pomegranate exocarp ethanolic extract as it displayed the higher antimicrobial and antioxidant activity (Fig.7). The cytotoxic activity of red, yellow, and purple pomegranate extracts against Huh7it cells was evaluated using the MTT assay”. This sentence should be clarified. The authors first refer to the “exocarp ethanolic extract” and then to the “cytotoxic activity of red, yellow, and purple pomegranate extracts”. This creates confusion, and the same issue recurs throughout the Results section. The authors should carefully check and revise the text to avoid ambiguity, clearly specifying which extract, fruit part, and variety are being discussed. In addition, whenever differences are reported, the authors should state whether these differences are statistically significant. | We sincerely thank the reviewer for highlighting this ambiguity regarding tissue and extract nomenclature, as well as the need for explicit statistical verification. We have thoroughly revised the text throughout the Results section to ensure absolute clarity and precision:   1. Resolution of Ambiguity (Specific Matrix Nomenclature): We have checked and revised the entire section to avoid generalized terms like *"pomegranate extracts"* or *"peel"*. The text has been updated to explicitly state that the assay was performed on the "pomegranate exocarp ethanolic extract " and specifies the precise cultivar comparisons (red, yellow, and purple varieties) for every data point mentioned. 2. Integration of Statistical Significance: In accordance with your guidance, we have incorporated the statistical parameters directly into the narrative. We have explicitly stated that the variations in antiproliferative potencies and IC50 shifts among the three cultivars were evaluated using One-way ANOVA followed by post-hoc testing, confirming that the reported differences are statistically significant (p < 0.05). Furthermore, standard deviations (SD) have been appended to each reported IC50 value.   The fully clarified and statistically validated paragraphs can be found in Lines [695-718] of the revised manuscript. |
| 46 | L528-542:  The LC-HRMS results and their interpretation require substantial clarification. The authors report valpromide as the compound with the largest peak area and describe it as an “artifact/contaminant candidate”, but it is then included among the major LC-HRMS identifications. Please clarify how this compound was identified and why it is reported as a major component of the extract if it may represent an artifact or contaminant. | We sincerely thank the reviewer for highlighting this inconsistency. We completely agree that including an external contaminant or analytical artifact in the primary list of plant secondary metabolites without adequate contextual isolation is misleading. We have thoroughly revised the text and data presentation to clarify how the compounds were identified and to properly isolate this non-plant feature:   1. Clarification of the Identification Workflow: We have clarified the methodology in the revised text. Metabolite annotation was performed using Compound Discoverer 3.2, based on high-resolution accurate mass measurements and MS/MS spectral library matching. Because authentic reference standards were not utilized, all assignments are strictly regarded as tentative annotations. 2. Justification of Valpromide as a Contaminant: Valpromide (C_9_H_19_NO) is a synthetic pharmacological agent and is not biogenetically synthesized by *Punica granatum*. Its appearance as the largest chromatographic peak (area ~12.2%) is attributed to a false-positive library match by the software algorithm or a procedural laboratory contaminant (such as micro-contamination from plasticware or chemical reagents). 3. Restructuring the Text Narrative: In the revised manuscript, we have strictly isolated valpromide from the *bona fide* phytochemical results. We explicitly state its nature as an artifact to maintain absolute data transparency, but immediately clarify that if this non-plant artifact is excluded, the true phytochemical profile of the purple pomegranate exocarp is completely dominated by target phenolic acids, flavonols (myricetin and quercetin derivatives), and ellagic acid.   The corrected paragraph reflecting this rigorous separation and improved terminology can be found in Lines [735-738] of the revised manuscript. |
| 47 | L531:  methanolic?????????? | We sincerely apologize for this critical typographical error. The word "methanolic" was accidentally introduced during drafting and has been corrected to "ethanolic" in the revised manuscript (Line [724]) to accurately reflect the 96% ethanol solvent utilized for this extraction group. We have double-checked the entire manuscript to ensure that all solvent designations are consistent and correct. |
| 48 | L549-564:  The GC-MS section requires clarification. The authors describe the analysis as the identification of phenolic compounds in the n-hexane extract, but the reported profile is dominated mainly by non-polar hydrocarbons, alkenes, fatty acids/esters, and related lipophilic compounds. This terminology should therefore be corrected. In addition, the authors should clarify whether the cited references directly support the contribution of the specific detected compounds to antioxidant activity. The antioxidant potential of pomegranate peel is generally associated mainly with polar phenolics, including ellagitannins, ellagic acid, gallic acid, flavonoids, and anthocyanin derivatives. The role of compounds such as 1-nonadecene, cetene, octacosane, dotriacontane, and low-abundance oleic acid should not be overinterpreted without additional evidence. Particular caution is also needed for 2,4-di-tert-butylphenol, which may occur as a natural compound but can also appear as a GC-MS background contaminant or artefact. The authors should report whether solvent, procedural, and extraction blanks were analysed, and whether compound identities were confirmed using retention indices, mass spectral match scores, authentic standards, or only tentative NIST library matching. Without these details, the link between the GC-MS profile and the antioxidant activity of the n-hexane extract remains speculative. | We sincerely appreciate the reviewer’s rigorous and deeply insightful critique on our GC-MS section. We agree that our initial drafting required stronger scientific caution regarding terminology, contamination controls, and bioactivity interpretations. We have thoroughly revised the text and data presentation to address all your concerns as follows:   1. Correction of Terminology: We have corrected the misleading opening statement. The term *"phenolic compounds"* has been replaced with *"phytochemical compounds"* to accurately represent the true nature of the *n*-hexane fraction, which is dominated by hydrocarbons, alkenes, and fatty acids rather than phenolics. 2. Clarification of Identification Confidence (Tentative Status): In alignment with your comment, we have explicitly stated in the revised text that because authentic reference standards and retention indices (RI) were not employed, all compound annotations must be strictly regarded as tentative, based solely on mass spectral match scores (SI and RSI) and probability percentages from the NIST library. 3. Data Transparency and Supplementary Material: To ensure absolute transparency without cluttering the main text, the complete GC-MS dataset—including the exact Retention Times (RT) CAS numbers, SI, RSI, Probability (%), and Relative Area (%) for all 26 tentatively annotated compounds—has been compiled and provided in the Supplementary Data (S4 Data). Meanwhile, the main manuscript text has been streamlined to only highlight the predominant compound classes. 4. Mitigating Overinterpretation of Antioxidant Activity: We agree that the weak antioxidant capacity IC50 of the *n*-hexane fraction is fundamentally due to the absolute predominance of non-polar lipophilic compounds. We have toned down the discussion to avoid overinterpreting the roles of 1-nonadecene, cetene, octacosane, and dotriacontane. These long-chain lipophilic compounds are now presented objectively as major structural components of the wax/lipid matrix of the mesocarp, without speculative claims regarding their direct radical scavenging contributions.   The completely revamped paragraph, including the corrected terminology and identification caveats, can be found in Lines [749-771] of the revised manuscript, and the raw analytical metrics are accessible in the Supplementary Data (S4 Data). |
| 49 | L557-563:  the authors should discuss the data in discussion section. | We completely agree with the reviewer’s structural guidance. In the original draft, scientific interpretations and discussions regarding the proximate parameters (such as microbial susceptibility, mineral functions, carbohydrate-phenolic complexes, etc.) were incorrectly placed within the Results section.  To adhere strictly to the standard journal layout, we have thoroughly reorganized this presentation:  In the Results section (Lines [774-788]): We have rewritten the text to focus exclusively on the objective, factual presentation of the raw proximate data (Table 3) and the data-driven justification for selecting the purple pomegranate exocarp for this analysis. |
| 50 | L610:  Pearson correlation….??? | We completely agree with the reviewer’s comment regarding the inconsistency of mentioning the Pearson correlation analysis without defining it within our methodology.  Upon re-evaluating the manuscript's statistical framework, we determined that our primary conclusions are already robustly supported by the comparative analysis of variance (ANOVA) and the explicit regression models used for IC_50_ derivation. Therefore, to streamline the findings and ensure absolute consistency between the methodology and the results, we have completely removed the statement regarding the Pearson correlation analysis from the manuscript including discussion section. |
| 51 | Several inconsistencies should be corrected throughout the Discussion. For example, the authors first state that ethanolic exocarp extracts showed the highest TPC, TFC and antioxidant activity, but later state that TPC, TFC and antioxidant capacity were higher in the mesocarp. These statements appear contradictory and should be reconciled using the actual data and statistical comparisons. | We sincerely thank the reviewer for pointing out this apparent contradiction. Upon re-examining our data, we clarify that the two statements refer to different solvent extracts and are therefore not contradictory: exocarp showed the highest TPC, TFC, and antioxidant activity in the ethanolic extract, whereas mesocarp showed higher TPC, TFC, and antioxidant capacity in the n-hexane extract. We have revised the Discussion to explicitly state the solvent context for each comparison, so that the two findings are presented as distinct patterns rather than conflicting claims. |
| 52 | Whenever differences among varieties, solvents, or fruit parts are discussed, the authors should clearly state whether these differences are statistically significant. | We appreciate this important comment. We have revised the Discussion so that every comparative statement (between solvents, fruit parts, or varieties) is now accompanied by a statement of statistical significance and the corresponding p-value, consistent with the statistical tests reported in the Results section. |
| 53 | The interpretation of the LC-HRMS and GC-MS data is problematic. The discussion does not clearly distinguish between the ethanolic purple exocarp analysed by LC-HRMS and the n-hexane red mesocarp analysed by GC-MS. Moreover, the major LC-HRMS compounds reported do not fully correspond to the typical phenolic markers expected in pomegranate peel. The authors should discuss these discrepancies more critically and provide identification confidence criteria before linking the detected compounds to bioactivity. | We thank the reviewer for this critical observation. We have restructured this part of the Discussion into two clearly separated sub-sections, explicitly identifying that LC-HRMS was performed on the ethanolic purple exocarp extract and GC-MS on the n-hexane red mesocarp extract, so that the two chemical profiles are no longer conflated. We have also added a discussion of why the major LC-HRMS compounds (D-(+)-Pyroglutamic acid and triacetonamine) do not correspond to the typical phenolic markers of pomegranate peel, along with the identification confidence criteria that should underpin any bioactivity linkage. |
| 54 | Statistical differences are indicated in the figure, but they are not described or discussed in the Results section. The authors should report the main statistical findings in the text and provide the corresponding p-values where appropriate. | We thank the reviewer for highlighting this gap. This comment pertains primarily to the Results section; we have ensured the Discussion is written to be fully consistent with the statistical findings that will be explicitly reported in the revised Results section |
| 55 | Covered under "several inconsistencies... throughout the Discussion | While revising the Discussion, we also identified and corrected an additional inconsistency: our results reported relatively weak antibacterial activity (IC50 ≥ 1000 µg/mL), yet the original text stated this finding was "aligned" with Yassin et al. (2021), who reported the extracts as highly effective. We have clarified that the agreement with Yassin et al. (2021) pertains specifically to the pattern of greater susceptibility of gram-positive over gram-negative bacteria, not to the overall potency of the antibacterial activity, and noted that the difference in potency is likely attributable to differences in extraction solvent and testing methodology.  The text has been updated as follows (Lines [909-917]) |
| 56 | Covered under "several inconsistencies... throughout the Discussion | We also noted that the opening paragraph originally implied that LC-HRMS, GC-MS, and proximate analysis were performed on a single "most promising antioxidant extract," which was inconsistent with the fact that two separate extracts (ethanolic exocarp analysed by LC-HRMS, and n-hexane mesocarp analysed by GC-MS) were actually used. We have revised this sentence for clarity and consistency with the rest of the Discussion.  The text has been updated as follows (Lines [936-939]) |
