# Supplementary material for: Comparative phytochemical profiling and bioactivity evaluation of three Indonesian pomegranate (Punica granatum) peel varieties: Antioxidant, antibacterial, and in vitro antiproliferative activities
Source: PLoS One. 2026 Sep 15;21(9):e0357371. doi: 10.1371/journal.pone.0357371 (PMC13577413; doi:10.1371/journal.pone.0357371)
Supplement: Peer review history

# Peer review: Decision Letter 0

**DOI:** 10.1371/journal.pone.0357371.r001

**Authors:** Shengwei Sun

25 May 2026

Dear Dr. SAPUTRO,

Thank you for submitting your manuscript to PLOS ONE. After careful consideration, we feel that it has merit but does not fully meet PLOS ONE’s publication criteria as it currently stands. Therefore, we invite you to submit a revised version of the manuscript that addresses the points raised during the review process.

Please submit your revised manuscript by Jul 09 2026 11:59PM. If you will need more time than this to complete your revisions, please reply to this message or contact the journal office at plosone@plos.org. When you're ready to submit your revision, log on to https://www.editorialmanager.com/pone/ and select the 'Submissions Needing Revision' folder to locate your manuscript file.

- A letter that responds to each point raised by the academic editor and reviewer(s). You should upload this letter as a separate file labeled 'Response to Reviewers'.
- A marked-up copy of your manuscript that highlights changes made to the original version. You should upload this as a separate file labeled 'Revised Manuscript with Track Changes'.
- An unmarked version of your revised paper without tracked changes. You should upload this as a separate file labeled 'Manuscript'.

If you would like to make changes to your financial disclosure, please include your updated statement in your cover letter. Guidelines for resubmitting your figure files are available below the reviewer comments at the end of this letter.

If applicable, we recommend that you deposit your laboratory protocols in protocols.io to enhance the reproducibility of your results. Protocols.io assigns your protocol its own identifier (DOI) so that it can be cited independently in the future. For instructions see: https://journals.plos.org/plosone/s/submission-guidelines#loc-laboratory-protocols. Additionally, PLOS ONE offers an option for publishing peer-reviewed Lab Protocol articles, which describe protocols hosted on protocols.io. Read more information on sharing protocols at https://plos.org/protocols?utm_medium=editorial-email&utm_source=authorletters&utm_campaign=protocols.

As the corresponding author, your ORCID iD is verified in the submission system and will appear in the published article. PLOS supports the use of ORCID, and we encourage all coauthors to register for an ORCID iD and use it as well. Please encourage your coauthors to verify their ORCID iD within the submission system before final acceptance, as unverified ORCID iDs will not appear in the published article. *Only*  the individual author can complete the verification step; PLOS staff *cannot*  verify ORCID iDs on behalf of authors.

We look forward to receiving your revised manuscript.

Kind regards,

Shengwei Sun, Ph.D.

Academic Editor

PLOS One

Journal Requirements:

When submitting your revision, we need you to address these additional requirements.

1.Please ensure that your manuscript meets PLOS ONE's style requirements, including those for file naming. The PLOS ONE style templates can be found at

https://journals.plos.org/plosone/s/file?id=wjVg/PLOSOne_formatting_sample_main_body.pdf and

https://journals.plos.org/plosone/s/file?id=ba62/PLOSOne_formatting_sample_title_authors_affiliations.pdf

2. In your Methods section, please provide details of the source of the pomegranates used in the study, including market location or company name. If you know the details of the cultivars, please include them.

3. Please provide a complete Data Availability Statement in the submission form, ensuring you include all necessary access information or a reason for why you are unable to make your data freely accessible. If your research concerns only data provided within your submission, please write "All data are in the manuscript and/or supporting information files" as your Data Availability Statement.

4. We note you have included a table to which you do not refer in the text of your manuscript. Please ensure that you refer to Table 1, 2 and 3 in your text; if accepted, production will need this reference to link the reader to the Table.

5. If the reviewer comments include a recommendation to cite specific previously published works, please review and evaluate these publications to determine whether they are relevant and should be cited. There is no requirement to cite these works unless the editor has indicated otherwise.

[Note: HTML markup is below. Please do not edit.]

Reviewer's Responses to Questions

**Comments to the Author**

1. Is the manuscript technically sound, and do the data support the conclusions?

Reviewer #1: Partly

**********

2. Has the statistical analysis been performed appropriately and rigorously?

Reviewer #1: No

**********

3. Have the authors made all data underlying the findings in their manuscript fully available?

The PLOS Data policy

Reviewer #1: No

**********

4. Is the manuscript presented in an intelligible fashion and written in standard English?

Reviewer #1: No

**********

Reviewer #1: General comments

The manuscript entitled “Comparative Phytochemical Profiling and Bioactivity Evaluation of Three Indonesian Pomegranate (Punica granatum) Peel Variety: Antioxidant, Antibacterial, and In vitro Antiproliferative Activities” addresses a relevant topic and may provide useful regional comparative data on Indonesian pomegranate varieties. However, the novelty should be presented more cautiously, as pomegranate peel is already well established as a source of antioxidant compounds. The manuscript would benefit from a clearer framing as a comparative phytochemical and bioactivity study, together with stronger support for LC-HRMS compound identification and a more appropriate statistical analysis. Provide means ± SD (or SE) and the number of biological and technical replicates for every quantitative result; include ANOVA/post hoc test details (assumptions checked, exact p values). Furthermore the Methods and Results alternately state that LC HRMS was performed on the “ethanol 96% extract” and that GC MS was performed on the “n hexane extract”, yet several bioassays and identification statements refer generically to “the extract” or to different solvents in different sections. For example: “The identification of bioactive compounds in the best performing antioxidant from the ethanol 96% extract was performed using Liquid Chromatography–High Resolution Mass Spectrometry (LC HRMS).” And later: “The chemical analysis of the best performing antioxidant from the n hexane extract of pomegranate peel was performed using GC MS.” Please harmonize the text so that each assay explicitly states which extract (solvent, tissue, and variety) was used and why that extract was selected for that analysis. The manuscript appears to analyse only the “best performing” extract in some downstream assays (LC HRMS, GC MS, proximate analysis) but does not justify the selection criteria or indicate whether representative replicates were profiled. State the objective criteria used to choose samples for chemical profiling and whether profiling was performed on pooled or individual replicates. For the reasons outlined above, in my opinion the Materials and Methods section requires substantial revision for clarity, accuracy, and reproducibility. Moreover, the Results section should be largely rewritten, as the findings are currently not presented in a clear and well-structured manner. Several paragraphs include generic background descriptions rather than direct results, and the quantitative outcomes are not always linked to appropriate statistical support. The authors should revise this section to present only the experimental findings, organize them logically, and clearly distinguish data interpretation from general discussion.

The Discussion section requires substantial rewriting. In its current form, it is largely descriptive and repetitive, and several paragraphs read as background information or a repetition of the Results rather than a critical interpretation of the findings. The authors should reorganize the Discussion around the main experimental outcomes: antioxidant capacity, antibacterial activity, antiproliferative activity, and phytochemical profiling. Each point should be interpreted in relation to the statistical results and compared critically with the literature. Overall, the Discussion should be extensively rewritten to improve clarity, coherence, and scientific rigor. The authors should remove generic background statements, avoid repeating the Results, resolve internal contradictions, provide statistical support for reported differences, and interpret the findings more cautiously.

L60–62:The statement comparing the present results with Andriani’s study is not sufficiently supported. The manuscript compares “purple pomegranate” with “black pomegranate” without clarifying whether these terms refer to the same cultivar/color category or to distinct genotypes. This is relevant because antioxidant activity in pomegranate peel is strongly influenced by cultivar identity and phytochemical composition, not simply by peel colour. Without clarification of cultivar equivalence and methodological comparability, the reported discrepancy with Andriani’s findings remains descriptive and may be misleading.

L63–81:The study objective should be stated more clearly and concisely. The final paragraph of the Introduction includes excessive methodological detail and should be shortened, leaving only the rationale and the specific aim of the work.

L90:The drying protocol for pomegranate peel should be described in greater detail. The authors should report the drying conditions, including ambient temperature, if available.

L113:This sentence should be moved to the Results section.

L116:The authors should use an appropriate term, such as “distilled water” or “deionized water”, and rephrase the sentence. For example: “A stock solution of the crude extract was prepared…”

L120–132:The calibration procedure is unclear. The concentrations reported as 100–3.125 µg mL⁻¹ appear to refer to the working gallic acid standards, not to the final reaction mixture. Since 0.20 mL of standard solution is diluted to a final volume of 4.20 mL, the final assay concentrations are 21-fold lower. The authors should clarify which concentrations were used to construct the calibration curve and how dilution factors were applied in the GAE calculation.

L144–148:See the comment above.

L155–156:Please check the sentence “standard curve of gallic acid”, as this section refers to quercetin standards for TFC determination.

L172–178:The method should be reported more clearly, using standard concentration units, preferably µg mL⁻¹ rather than ppm. The term “aquabides” should be replaced with double-distilled or deionized water. The rationale for preparing standards in both water and DMSO should also be explained, and appropriate solvent blanks should be included.

L199–214:The introductory description of the ABTS method should be revised or removed from the Materials and Methods section. The statement that ABTS is “also known as ABTS/TAC or ABTS/TEAC” is inaccurate: ABTS is the reagent/assay system, whereas TEAC refers to Trolox equivalent antioxidant capacity, and TAC is a broader analytical concept. In addition, ABTS itself is not “produced” by oxidation; rather, the ABTS radical cation, ABTS•⁺, is generated by reacting ABTS with potassium persulfate. Since the authors subsequently describe the actual reagent preparation, this paragraph should focus directly on the experimental protocol and avoid general textbook-style statements.

Moreover, there is an inconsistency between the use of a Trolox standard curve and the expression of results as IC₅₀. If results are expressed as IC₅₀, the authors must test a concentration series of each extract and calculate the concentration producing 50% inhibition. Conversely, if a Trolox calibration curve is used, results should be expressed as Trolox equivalents. The current description mixes the two approaches and should be clarified.

L232:The authors should clarify and correct the description of the IC₅₀ calculation. As written, the formula IC₅₀ = (50 − a) / b is only valid when the linear regression relates percentage inhibition directly to concentration. If the regression was performed using ln(concentration) as the predictor, the correct expression is IC₅₀ = exp[(50 − a) / b]. The authors should explicitly state which predictor was used and apply the corresponding formula.

L241:Please correct the typo “ThermoScientific™ Accucore™” to “Thermo Scientific™ Accucore™”, and check the manuscript for inconsistent brand formatting.

L243–245:The elution program is ambiguous. The authors should clarify it by providing explicit time points and hold durations, including initial conditions, gradient endpoint, hold period, return to initial conditions, and re-equilibration time.

L250:A single spray voltage is reported, although acquisition was performed in both positive and negative ion modes. The authors should specify the spray voltage used for each polarity and describe the polarity-switching strategy.

LC-HRMS identification criteria:The authors should specify the compound identification criteria, including mass tolerance, MS/MS matching thresholds, databases or libraries used, and whether authentic standards were employed for confirmation.

L256:Why was only the n-hexane extract selected for GC-MS analysis? The selection criterion should be clearly justified.

L267:Were authentic standards used to confirm compound identification? If not, the level of identification confidence should be clearly stated.

L277:The authors should provide additional information on the origin of the bacterial strains.

L279:The sentence “was examined for the pomegranate peel ethanolic extract” should be revised.

More importantly, the rationale for using ethanolic extracts in some assays and n-hexane extracts in others is unclear. In my opinion, the Materials and Methods section requires careful revision because several inconsistencies are present.

L289–293:OD measurements are reported at 630 nm and converted to CFU estimates. The bacterial inoculum appears very high, since OD₆₃₀ = 0.4 is reported to correspond to approximately 10⁸–10⁹ CFU mL⁻¹ before dilution. The authors should provide the basis for these conversions, such as a calibration curve or McFarland standard, and either use the more conventional OD₆₀₀ or explain why OD₆₃₀ was chosen. The actual CFU mL⁻¹ used in the assay and the number of biological replicates should also be reported.

L298–306:The authors should explain how the final extract concentrations were achieved in the wells, including stock concentration, dilution scheme, and final assay volume. The concentration range tested and the final solvent concentration in the wells should be reported. Although the authors state that DMSO did not exceed 1% v/v, the final well concentration should be explicitly confirmed. The concentration of the positive control, sorbic acid, should also be specified, and the rationale for its selection should be provided.

Sterility controls, vehicle controls, and extract blanks should be clearly described. Since plant extracts may cause turbidity or absorbance at the measurement wavelength, the authors should state how blank absorbance was handled to correct for extract colour or turbidity. Additional verification, such as viable counts or an alternative viability assay, would strengthen the reliability of the OD-based measurements.

L322:Please correct the typo “cacer cells”.

L339–341:The statistical analysis is insufficiently described. The authors should clearly specify the variables analysed, the experimental unit, the number and type of replicates, the significance threshold, and whether ANOVA assumptions were checked. The use of Duncan’s test should also be justified, or replaced with a more appropriate multiple-comparison procedure. As currently written, the statistical approach is too generic and not fully reproducible.

L358–369:This section reads as background information rather than experimental results. Unless the authors performed anatomical or histological analyses, the description of exocarp and mesocarp tissues is unnecessary in the Results section and should be removed or relocated. The Results section should focus on the data generated in this study.

L382–383:Pearson correlation analysis is mentioned in the Results, but it is not described in the Materials and Methods section. The authors should either describe this analysis in the statistical methods or remove the statement.

L388–389:When reporting significant effects, the authors should provide the corresponding p-values. This should be checked and revised throughout the manuscript.

L442 and L448:These interpretative comments should be moved to the Discussion section.

L460:Please correct the typo “$”.

L462–465:This sentence should be checked and revised for clarity.

L467:The sentence “Overall, the ethanolic peel extracts demonstrated stronger activity than their mesocarp counterparts” should be checked and revised. The authors may mean that ethanolic exocarp extracts showed stronger activity than ethanolic mesocarp extracts, but this needs to be clearly stated. The paragraph should be carefully revised to distinguish between exocarp and mesocarp extracts.

L482–483: Please clarify this point. Why did the authors not determine the minimum inhibitory concentration (MIC)? MIC is one of the most widely used parameters for assessing antimicrobial activity and is generally more informative from an applicative perspective, as it indicates the concentration required to inhibit visible bacterial growth. Another relevant parameter is the minimum bactericidal concentration (MBC). Did the authors evaluate MBC as well?

L488-491: n this section, the authors should report only the results. Interpretation and discussion of the findings should be moved to the Discussion section.

L507: “The antiproliferative efficiency of pomegranate peel against cancer cells was examined for the pomegranate exocarp ethanolic extract as it displayed the higher antimicrobial and antioxidant activity (Fig.7). The cytotoxic activity of red, yellow, and purple pomegranate extracts against Huh7it cells was evaluated using the MTT assay”. This sentence should be clarified. The authors first refer to the “exocarp ethanolic extract” and then to the “cytotoxic activity of red, yellow, and purple pomegranate extracts”. This creates confusion, and the same issue recurs throughout the Results section. The authors should carefully check and revise the text to avoid ambiguity, clearly specifying which extract, fruit part, and variety are being discussed. In addition, whenever differences are reported, the authors should state whether these differences are statistically significant.

L528-542: The LC-HRMS results and their interpretation require substantial clarification. The authors report valpromide as the compound with the largest peak area and describe it as an “artifact/contaminant candidate”, but it is then included among the major LC-HRMS identifications. Please clarify how this compound was identified and why it is reported as a major component of the extract if it may represent an artifact or contaminant.

L531: methanolic??????????

L549-564: The GC-MS section requires clarification. The authors describe the analysis as the identification of phenolic compounds in the n-hexane extract, but the reported profile is dominated mainly by non-polar hydrocarbons, alkenes, fatty acids/esters, and related lipophilic compounds. This terminology should therefore be corrected. In addition, the authors should clarify whether the cited references directly support the contribution of the specific detected compounds to antioxidant activity. The antioxidant potential of pomegranate peel is generally associated mainly with polar phenolics, including ellagitannins, ellagic acid, gallic acid, flavonoids, and anthocyanin derivatives. The role of compounds such as 1-nonadecene, cetene, octacosane, dotriacontane, and low-abundance oleic acid should not be overinterpreted without additional evidence. Particular caution is also needed for 2,4-di-tert-butylphenol, which may occur as a natural compound but can also appear as a GC-MS background contaminant or artefact. The authors should report whether solvent, procedural, and extraction blanks were analysed, and whether compound identities were confirmed using retention indices, mass spectral match scores, authentic standards, or only tentative NIST library matching. Without these details, the link between the GC-MS profile and the antioxidant activity of the n-hexane extract remains speculative.

L557-563:the authors should discuss the data in discussion section.

L610: Pearson correlation….???

Discussion

Several inconsistencies should be corrected throughout the Discussion. For example, the authors first state that ethanolic exocarp extracts showed the highest TPC, TFC and antioxidant activity, but later state that TPC, TFC and antioxidant capacity were higher in the mesocarp. These statements appear contradictory and should be reconciled using the actual data and statistical comparisons. Whenever differences among varieties, solvents, or fruit parts are discussed, the authors should clearly state whether these differences are statistically significant. The interpretation of the LC-HRMS and GC-MS data is problematic. The authors state that these analyses elucidate the chemical basis of antioxidant and cytotoxic activities, but the discussion does not clearly distinguish between the ethanolic purple exocarp analysed by LC-HRMS and the n-hexane red mesocarp analysed by GC-MS. Moreover, the major LC-HRMS compounds reported do not fully correspond to the typical phenolic markers expected in pomegranate peel, such as punicalagin, punicalin, gallic acid, ellagic acid derivatives, flavonoids, and anthocyanins. The authors should discuss these discrepancies more critically and provide identification confidence criteria before linking the detected compounds to bioactivity.

Statistical differences are indicated in the figure, but they are not described or discussed in the Results section. The authors should report the main statistical findings in the text and provide the corresponding p-values where appropriate.

**********

what does this mean?). If published, this will include your full peer review and any attached files.

If you choose “no”, your identity will remain anonymous but your review may still be made public.

**Do you want your identity to be public for this peer review?**  For information about this choice, including consent withdrawal, please see our Privacy Policy

Reviewer #1: No

**********

[NOTE: If reviewer comments were submitted as an attachment file, they will be attached to this email and accessible via the submission site. Please log into your account, locate the manuscript record, and check for the action link "View Attachments". If this link does not appear, there are no attachment files.]

To ensure your figures meet our technical requirements, please review our figure guidelines: https://journals.plos.org/plosone/s/figures

You may also use PLOS’s free figure tool, NAAS, to help you prepare publication quality figures: https://journals.plos.org/plosone/s/figures#loc-tools-for-figure-preparation.

NAAS will assess whether your figures meet our technical requirements by comparing each figure against our figure specifications.

# Author comment: Author response to Decision Letter 1

**DOI:** 10.1371/journal.pone.0357371.r002

7 Aug 2026

All the responses to reviewer were include in the file response to reviewer

Attachment Submitted filename: Response to Reviewers With tables Completed.docx

# Editor report: Decision Letter 1

**DOI:** 10.1371/journal.pone.0357371.r003

**Authors:** Shengwei Sun

17 Aug 2026

Comparative Phytochemical Profiling and Bioactivity Evaluation of Three Indonesian Pomegranate (Punica granatum) Peel Variety: Antioxidant, Antibacterial, and In vitro Antiproliferative Activities

PONE-D-26-19355R1

Dear Dr. SAPUTRO,

We’re pleased to inform you that your manuscript has been judged scientifically suitable for publication and will be formally accepted for publication once it meets all outstanding technical requirements.

Within one week, you’ll receive an e-mail detailing the required amendments. When these have been addressed, you’ll receive a formal acceptance letter and your manuscript will be scheduled for publication.

An invoice will be generated when your article is formally accepted. Please note, if your institution has a publishing partnership with PLOS and your article meets the relevant criteria, all or part of your publication costs will be covered. Please make sure your user information is up-to-date by logging into Editorial Manager at Editorial Manager® and clicking the ‘Update My Information' link at the top of the page. For questions related to billing, please contact billing support.

If your institution or institutions have a press office, please notify them about your upcoming paper to help maximize its impact. If they’ll be preparing press materials, please inform our press team as soon as possible -- no later than 48 hours after receiving the formal acceptance. Your manuscript will remain under strict press embargo until 2 pm Eastern Time on the date of publication. For more information, please contact onepress@plos.org.

Kind regards,

Shengwei Sun, Ph.D.

Academic Editor

PLOS One

Additional Editor Comments (optional):

Reviewers' comments:

# Editor report: Acceptance letter

**DOI:** 10.1371/journal.pone.0357371.r004

**Authors:** Shengwei Sun

PONE-D-26-19355R1

PLOS One

Dear Dr. Saputro,

I'm pleased to inform you that your manuscript has been deemed suitable for publication in PLOS One. Congratulations! Your manuscript is now being handed over to our production team.

At this stage, our production department will prepare your paper for publication. This includes ensuring the following:

* All references, tables, and figures are properly cited

* All relevant supporting information is included in the manuscript submission,

* There are no issues that prevent the paper from being properly typeset

You will receive further instructions from the production team, including instructions on how to review your proof when it is ready. Please keep in mind that we are working through a large volume of accepted articles, so please give us a few days to review your paper and let you know the next and final steps.

Lastly, if your institution or institutions have a press office, please let them know about your upcoming paper now to help maximize its impact. If they'll be preparing press materials, please inform our press team within the next 48 hours. Your manuscript will remain under strict press embargo until 2 pm Eastern Time on the date of publication. For more information, please contact onepress@plos.org.

You will receive an invoice from PLOS for your publication fee after your manuscript has reached the completed accept phase. If you receive an email requesting payment before acceptance or for any other service, this may be a phishing scheme. Learn how to identify phishing emails and protect your accounts at https://explore.plos.org/phishing.

If we can help with anything else, please email us at customercare@plos.org.

Thank you for submitting your work to PLOS One and supporting open access.

Kind regards,

PLOS One Editorial Office Staff

on behalf of

Dr. Shengwei Sun

Academic Editor

PLOS One
